# MiR‐103‐3p targets the m^6^A methyltransferase METTL14 to inhibit osteoblastic bone formation

**DOI:** 10.1111/acel.13298

**Published:** 2021-01-13

**Authors:** Zhongyang Sun, Han Wang, Yuxiang Wang, Guodong Yuan, Xin Yu, Hui Jiang, Qi Wu, Binkui Yang, Zebing Hu, Fei Shi, Xinsheng Cao, Shu Zhang, Ting Guo, Jianning Zhao

**Affiliations:** ^1^ Department of Orthopedics, Affiliated Jinling Hospital Medical School of Nanjing University Nanjing China; ^2^ Department of Orthopedics, Air Force Hospital of Eastern Theater Anhui Medical University Nanjing China; ^3^ Department of Orthopedics Air Force Medical Center Beijing China; ^4^ Medical School of Southeast University Nanjing China; ^5^ Hangzhou Special Sanatorium Center of the PLA Air Force Nanjing China; ^6^ The Key Laboratory of Aerospace Medicine, Ministry of Education Air Force Medical University Xi'an China

**Keywords:** METTL14, miR‐103‐3p, N^6^‐methyladenosine, osteoblast activity, osteoporosis

## Abstract

Impaired osteoblast function is involved in osteoporosis, and microRNA (miRNA) dysregulation may cause abnormal osteoblast osteogenic activity. However, the influence of miRNA on osteoblast activity and the underlying mechanisms remain elusive. In this study, miR‐103‐3p was found to be negatively correlated with bone formation in bone specimens from elderly women with fractures and ovariectomized (OVX) mice. Additionally, miR‐103‐3p directly targeted *Mettl14* to inhibit osteoblast activity, and METTL14‐dependent N^6^‐methyladenosine (m^6^A) methylation inhibited miR‐103‐3p processing by the microprocessor protein DGCR8 and promoted osteoblast activity. Moreover, miR‐103‐3p inhibited bone formation in vivo, and therapeutic inhibition of miR‐103‐3p counteracted the decreased bone formation in OVX mice. Further, *METTL14* was negatively correlated with miR‐103‐3p but positively correlated with bone formation in bone specimens from elderly women with fractures and OVX mice. Collectively, our results highlight the critical roles of the miR‐103‐3p/METTL14/m^6^A signaling axis in osteoblast activity, identifying this axis as a potential target for ameliorating osteoporosis.

## Graphical abstract

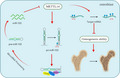

## 1 INTRODUCTION

Osteoporosis is a common disease among elderly women and is characterized by reduced bone mass and abnormal microarchitecture, resulting in fragility fractures (Rachner et al., 2011). Emerging evidence indicates that osteoblasts directly affect the entire bone remodeling process and that impaired osteoblast activity plays fundamental roles in bone metabolic disorder (Harada, & Rodan, 2003). Moreover, the mechanisms that mediate impaired osteoblast activity remain unclear and merit further research.

microRNAs (miRNAs) are small single‐stranded noncoding RNA molecules involved in silencing and post‐transcriptional regulation of gene expression, thereby mediating many biological processes (Rigoutsos, & Furnari, 2010; Sun, & Lai, 2013). Many miRNAs have been characterized to regulate osteoblast activity and osteoblastic bone formation (Inose et al., 2009; Wang et al., 2013; Xu et al., 2018). Our previous study revealed that miR‐103‐3p could inhibit L‐type calcium channel currents and osteoblast proliferation under simulated microgravity conditions, primarily by suppressing Cav1.2 expression (Sun, Cao, Hu, et al., 2015; Sun, Cao, Zhang, et al., 2015). Other researchers showed that miR‐103‐3p was downregulated under cyclic mechanical loading conditions and found that this alteration was adversely correlated with osteoblast differentiation and bone formation in response to mechanical stimulation (Zuo et al., 2015). In addition, we found that mature miR‐103‐3p was evolutionarily conserved among several species and highly expressed in bone tissue (Figure S1a,b). However, the influence of miR‐103‐3p on osteoblastic bone formation in postmenopausal osteoporosis and the possible underlying mechanisms have not been confirmed.

N^6^‐methyladenosine (m^6^A) is the most prevalent epigenetic modification in eukaryotic mRNAs and regulates a broad spectrum of biological processes (Wang, & He, 2014; Zhao, & He, 2015; Zhao et al., 2017). In mammals, m^6^A is installed by an m^6^A methyltransferase complex consisting of methyltransferase‐like 3 (METTL3), methyltransferase‐like 14 (METTL14), and Wilms tumor 1‐associated protein (WTAP) and can be removed by the demethylases alkB homologue 5 (ALKBH5) and fat mass and obesity‐associated protein (FTO) (Fu et al., 2014; Yue et al., 2015). The m^6^A methyltransferase METTL3 (Tian et al., 2019; Wu et al., 2018; Yao et al., 2019; Yu et al., 2020; Zhang et al., 2020) and demethylase FTO (Guo et al., 2011; Li et al., 2019; Sachse et al., 2018; Shen et al., 2018; Zhang, Riddle, et al., 2019) have been reported to regulate osteogenic activity and bone formation. However, the biological significance of the other m^6^A modulators in bone formation or osteoporosis remains elusive.

In this study, we tested the expression of miR‐103‐3p and bone formation marker genes in bone specimens from elderly women with fractures and in OVX mice and found that miR‐103‐3p was negatively correlated with bone formation. We present in vitro evidence demonstrating that miR‐103‐3p directly targets *Mettl14* to functionally inhibit osteoblast activity and that METTL14‐dependent m^6^A methylation provides negative feedback to regulate miR‐103‐3p processing by the microprocessor protein DGCR8, thereby modulating osteoblast activity. Moreover, we demonstrate that miR‐103‐3p inhibits bone formation in vivo and that therapeutic inhibition of miR‐103‐3p counteracts the decrease in bone formation in OVX mice. Further, we confirmed that *METTL14* is negatively correlated with miR‐103‐3p but positively correlated with bone formation in bone specimens from elderly women with fractures and in OVX mice.

## 2 RESULTS

### 2.1 High miR‐103‐3p expression is correlated with reduced bone formation capacity

**FIGURE 1:**
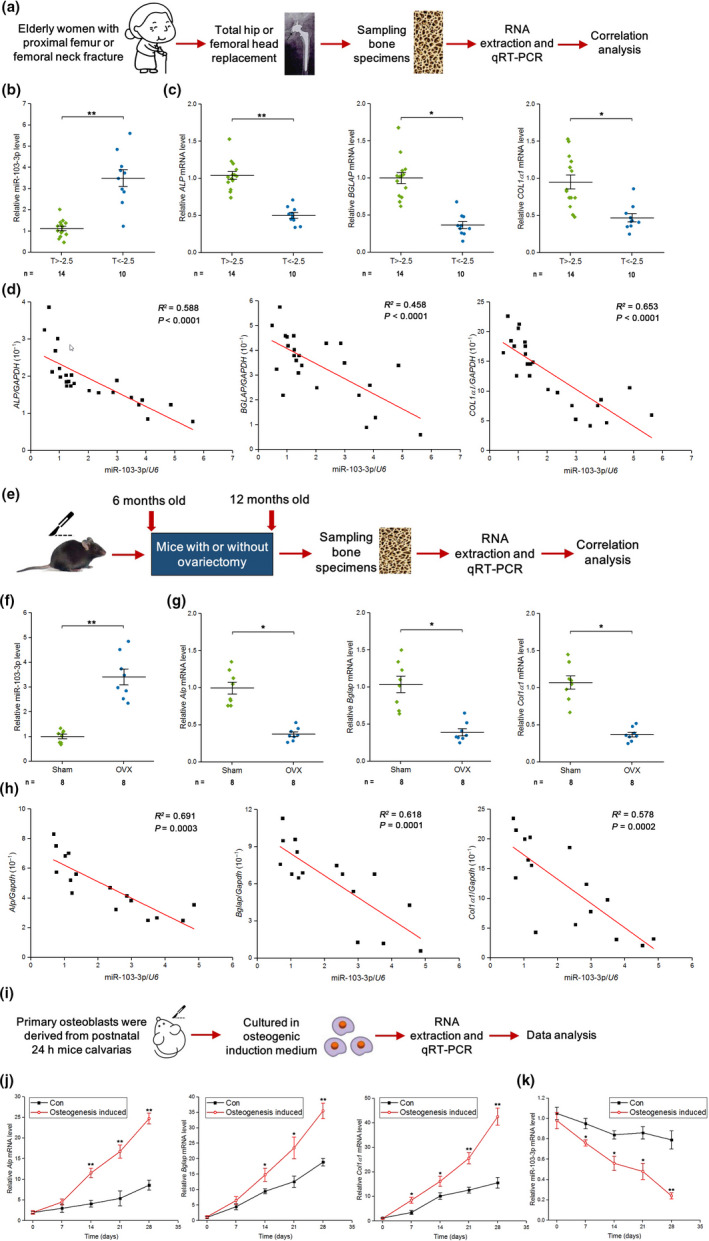
miR‐103‐3p levels are negatively correlated with bone formation capacity in human bone specimens, mouse bone specimens, and primary mouse osteoblasts. (a) Schematic diagram illustrating the experimental design. (b) Real‐time PCR analysis of the bone mineral density‐related changes in miR‐103‐3p levels in bone specimens from patients. The relative miRNA levels were normalized to the mean value of the T > −2.5 group. *U6* small nuclear RNA was used as the internal control. (c) Real‐time PCR analysis of the bone mineral density‐related changes in the mRNA levels of the bone formation marker genes *ALP* (left), *BGLAP* (middle), and *COL1α1* (right) in bone specimens from patients. The relative mRNA levels were normalized to the mean value of the T > −2.5 group. Human *GAPDH* mRNA was used as the internal control. (d) Correlation analysis between the miR‐103‐3p level and *ALP* (left), *BGLAP* (middle), and *COL1α1* (right) mRNA levels in bone specimens from patients. (e) Schematic diagram illustrating the experimental design. (f) Real‐time PCR analysis of the changes in miR‐103‐3p levels in bone specimens from Sham or OVX mice. The relative miRNA levels were normalized to the mean value of the Sham group. *U6* small nuclear RNA was used as the internal control. (g) Real‐time PCR analysis of the changes in the mRNA levels of the bone formation marker genes *Alp* (left), *Bglap* (middle) and *Col1α1* (right) in bone specimens from Sham or OVX mice. The relative mRNA levels were normalized to the mean value of the Sham group. Mouse *Gapdh* mRNA was used as the internal control. (h) Correlation analysis between the miR‐103‐3p level and *Alp* (left), *Bglap* (middle), and *Col1α1* (right) mRNA levels in bone specimens from Sham or OVX mice. (i) Schematic diagram illustrating the experimental design. (j) Real‐time PCR analysis of *Alp* (left), *Bglap* (middle), and *Col1α1* (right) mRNA levels in primary mouse osteoblasts during osteoblast maturation (*n* = 4). (k) Real‐time PCR analysis of miR‐103‐3p levels in primary mouse osteoblasts during osteoblast maturation (*n* = 4). The *n* value for each group is indicated at the bottom of each bar in the graphs. All data are the mean ± *SD*. *p < 0.05, **p < 0.01. One‐way analysis of variance (ANOVA) with a post hoc test was performed, and the significance of differences between two groups was determined with Student's *t* test. For statistical correlation, Pearson's correlation coefficient was used

To examine the expression pattern of miR‐103‐3p and bone formation marker genes in human bone tissues, we collected bone specimens from 24 elderly female patients with fractures (Figure 1a and Tables S1 and S2) and examined miR‐103‐3p, *ALP* (alkaline phosphatase), *BGLAP* (osteocalcin), and *COL1α1* (collagen type 1α1) expression in these bone specimens by q‐PCR. The q‐PCR analysis showed that miR‐103‐3p levels in osteoporosis patients (T ≤ −2.5) were higher than in control patients (T > −2.5) (Figure 1b) and that the levels of *ALP*, *BGLAP*, and *COL1α1* in osteoporosis patients (T ≤ −2.5) were lower than in control patients (T > −2.5) (Figure 1c). In addition, miR‐103‐3p levels were negatively correlated with *ALP*, *BGLAP*, and *COL1α1* levels in these human samples (Figure 1d).

Mature miR‐103‐3p is evolutionarily conserved among several species (Figure S1a). Thus, we further investigated the relationship between the miR‐103‐3p level and bone formation marker genes in bone tissues from OVX mice (Figure 1e and Figure S1c‐e). We found high levels of miR‐103‐3p and low levels of *Alp*, *Bglap*, and *Col1α1* in bone tissues from OVX mice (Figure 1f,g). Consistently, we found that miR‐103‐3p levels were negatively correlated with *Alp*, *Bglap*, and *Col1α1* levels in these mouse samples (Figure 1h).

To further verify the relationship between miR‐103‐3p and osteoblast differentiation marker genes in osteoblasts, we derived and cultured primary osteoblasts from postnatal mouse calvarias (Figure 1i and Figure S1f,g) and examined miR‐103‐3p, *Alp*, *Bglap*, and *Col1α1* expression in primary osteoblasts by q‐PCR. We found that the *Alp*, *Bglap*, and *Col1α1* levels gradually increased (Figure 1j) and the miR‐103‐3p level decreased with osteoblast differentiation (Figure 1k). We found a similar trend for miR‐103‐3p and osteoblast differentiation marker genes in human hFOB 1.19 cells (human osteoblast‐like cells) (Figure S1 h,i).

### 2.2 miR‐103‐3p inhibits osteoblast proliferation, differentiation, and matrix mineralization

**FIGURE 2:**
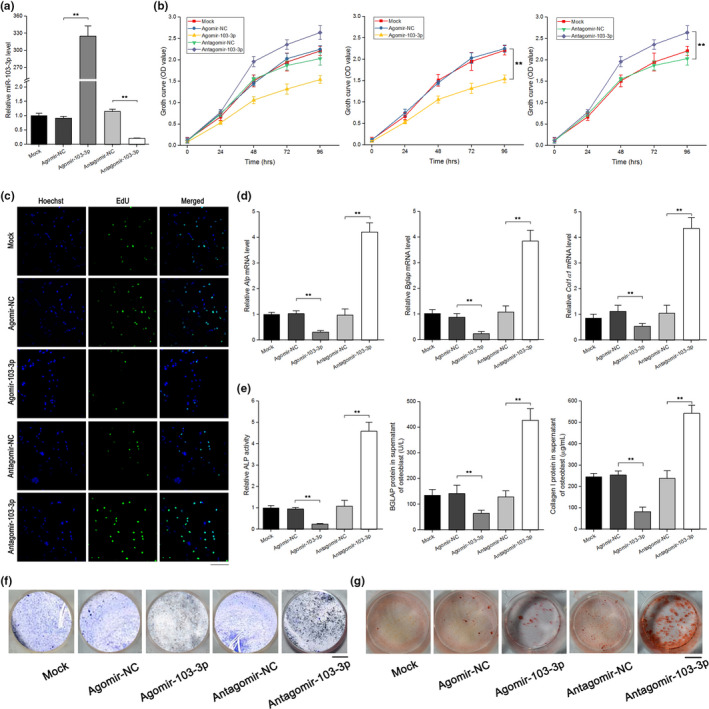
miR‐103‐3p inhibits osteoblast activity in vitro. (a) Real‐time PCR analysis of miR‐103‐3p levels in primary mouse osteoblasts after treatment with 300 μM agomir‐103‐3p, antagomir‐103‐3p, or the corresponding negative controls for 48 h (*n* = 3). (b) WST‐8 assay of changes in cell growth in each group at 24–96 h after treatment with 300 μM agomir‐103‐3p, antagomir‐103‐3p or the corresponding negative controls (*n* = 4). (c) EdU incorporation assay of the proliferation of primary mouse osteoblasts in each group after treatment with 300 μM agomir‐103‐3p, antagomir‐103‐3p or the corresponding negative controls for 48 h (n = 3). Cells were staining with the nucleic acid dye Hoechst (blue) and EdU (green). Scale bar, 10 μm. (d) Real‐time PCR analysis of the changes in the mRNA levels of the osteoblast differentiation marker genes *Alp* (left), *Bglap* (middle), and *Col1α1* (right) in primary mouse osteoblasts after treatment with 300 μM agomir‐103‐3p, antagomir‐103‐3p, or the corresponding negative controls for 48 h (*n* = 4). (e) ALP activity (left) and the amount of BGLAP protein (middle) and collagen I (right) in the supernatant of primary mouse osteoblasts after treatment with 300 μM agomir‐103‐3p, antagomir‐103‐3p, or the corresponding negative controls for 48 h (*n* = 4). (f) Representative images of ALP staining of primary mouse osteoblasts after treatment with 300 μM agomir‐103‐3p, antagomir‐103‐3p, or the corresponding negative controls for 48 h (*n* = 3). Scale bar, 10 mm. (g) Alizarin red staining of calcium deposition in primary mouse osteoblasts after treatment with 300 μM agomir‐103‐3p, antagomir‐103‐3p, or the corresponding negative controls in osteogenic medium for 21 days. Scale bar, 10 mm. All data are presented as the mean ± *SD*. **p < 0.01. One‐way ANOVA with a post hoc test was performed, and the significance of differences between two groups was determined with Student's *t* test

To determine the effects of miR‐103‐3p on osteoblast activity, we transfected primary osteoblasts with agomir‐103‐3p (a miR‐103‐3p agonist) or antagomir‐103‐3p (a miR‐103‐3p inhibitor) and then examined the efficiency of miR‐103‐3p overexpression and knockdown. Intracellular miR‐103‐3p levels were significantly upregulated by agomir‐103‐3p treatment and markedly downregulated by agomir‐103‐3p treatment (Figure 2a and Figure S2a).

To investigate the role of miR‐103‐3p in osteoblast proliferation, WST‐8 assays and EdU labeling assays were conducted. The time‐dependent growth curve was shifted downward in cells transfected with agomir‐103‐3p, whereas the growth curve was shifted upward in cells transfected with antagomir‐103‐3p compared to cells transfected with antagomir or agomir negative control (NC) (Figure 2b). EdU labeling assays showed that the growth of primary osteoblasts was significantly inhibited by agomir‐103‐3p relative to agomir‐NC. In addition, compared with antagomir‐NC, antagomir‐103‐3p enhanced the growth of osteoblasts (Figure 2c). We found a similar effect of miR‐103‐3p in hFOB 1.19 cells (Figure S2b).

To test the influence of miR‐103‐3p on osteoblast differentiation, we examined the levels of osteoblast differentiation markers and ALP staining in primary mouse osteoblasts. Compared to those in the corresponding control treatment groups, the *Alp*, *Bglap*, and *Col1α1* levels were upregulated by antagomir‐103‐3p and downregulated by agomir‐103‐3p (Figure 2d). ALP activity and the amount of BGLAP protein and collagen I in the supernatant of primary mouse osteoblasts were lower in the agomir‐103‐3p treatment group and higher in the antagomir‐103‐3p treatment group than in the agomir‐NC and antagomir‐NC treatment groups, respectively (Figure 2e). ALP staining assays showed that agomir‐103‐3p reduced ALP staining in primary mouse osteoblasts, whereas antagomir‐103‐3p enhanced ALP staining in the cells (Figure 2f). Similar data were also obtained in hFOB 1.19 cells (Figure S2c,d).

To examine the effects of miR‐103‐3p on osteoblast matrix mineralization, we performed an Alizarin red staining assay and demonstrated that agomir‐103‐3p decreased mineral deposition whereas antagomir‐103‐3p increased mineral deposition compared to that in the corresponding control treatment groups (Figure 2g). To determine the effects of miR‐103‐3p on osteoclast activity, an in vitro osteoclastogenesis assay was performed. The data revealed that miR‐103‐3p had no effect on osteoclast activity (Figure S2e–g).

### 2.3 miR‐103‐3p directly targets *Mettl14* to functionally inhibit osteoblast activity

**FIGURE 3:**
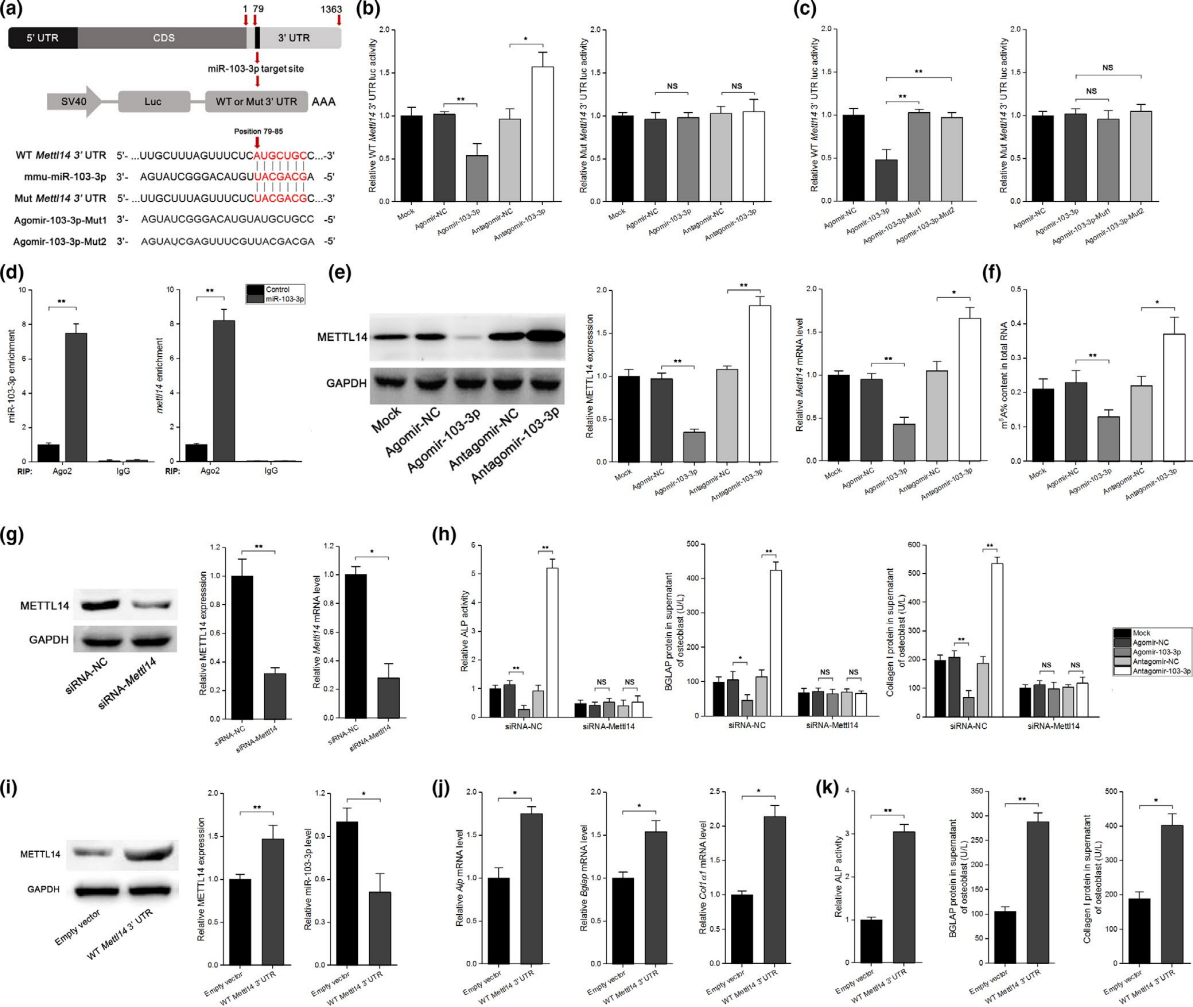
miR‐103‐3p directly targets *Mettl14* to inhibit the function of osteoblasts in vitro. (a) Schematic illustration of the design of luciferase reporters containing the WT *Mettl14* 3′UTR or the site‐directed mutant *Mettl14* 3’UTR. The pairing regions of miR‐103‐3p and the sequences of the two Agomir‐103‐3p mutants (Agomir‐103‐3p‐Mut1 and Agomir‐103‐3p‐Mut2) are also shown. (b) The effects of agomir‐103‐3p, antagomir‐103‐3p or the corresponding negative controls (agomir‐NC and antagomir‐NC) on luciferase activity in MC3 T3‐E1 cells transfected with either the WT *Mettl14* 3′UTR reporter (left) or the mutant *Mettl14* 3′UTR reporter (right) (*n* = 3). (c) The effect of agomir‐NC, agomir‐103‐3p, and mutated agomir‐103‐3p on luciferase activity in MC3 T3‐E1 cells transfected with either the WT *Mettl14* 3′UTR reporter (left) or the mutant *Mettl14* 3’UTR reporter (right) (*n* = 3). (d) RNA immunoprecipitation (RIP) assays showing the association with Ago2 of both miR‐103‐3p (left) and *Mettl14* mRNA (right) in MC3 T3‐E1 cells overexpressing miR‐103‐3p (*n* = 3). (e) The effect of agomir‐103‐3p, antagomir‐103‐3p, or the corresponding negative controls on the METTL14 protein (left and middle) and *Mettl14* mRNA (right) levels in primary mouse osteoblasts (*n* = 4). (f) The effect of agomir‐103‐3p, antagomir‐103‐3p, or the corresponding negative controls on the m^6^A content in total RNA (*n* = 3). (g) Western blot (left and middle) and real‐time PCR (right) analysis of the knockdown efficiency of *Mettl14*‐siRNA after treatment with a specific siRNA targeting *Mettl14* (siRNA‐*Mettl14*) or a nonspecific siRNA control (siRNA‐NC) (*n* = 3). (h) ALP activity (left) and the amount of BGLAP protein (middle) and collagen I (right) in the supernatant of primary mouse osteoblasts after silencing of *Mettl14* with siRNA‐*Mettl14* and treatment with agomir‐103‐3p, antagomir‐103‐3p, or the corresponding negative controls (*n* = 3). The results from siRNA‐NC and mock transfection are also shown. (i) Western blot (left and middle) and real‐time PCR (right) analysis of the effect of WT *Mettl14* 3′UTR on the METTL14 protein (left and middle) and miR‐103‐3p (right) levels in primary mouse osteoblasts (*n* = 3). (j) Real‐time PCR analysis of the changes in the mRNA levels of the osteoblast differentiation marker genes *Alp* (left), *Bglap* (middle), and *Col1α1* (right) in primary mouse osteoblasts after blockade of miR‐103‐3p binding to *Mettl14* via overexpression of WT *Mettl14* 3′UTR (*n* = 3). (k) ALP activity (left) and the amount of BGLAP protein (middle) and collagen I (right) in the supernatant of primary mouse osteoblasts after blockade of miR‐103‐3p binding to *Mettl14* via overexpression of WT *Mettl14* 3′UTR (*n* = 3). The results from the Empty vector and mock transfection are also shown. All data are the mean ± *SD*. *p < 0.05, **p < 0.01. One‐way ANOVA with a post hoc test was performed, and the significance of differences between two groups was determined with Student's *t* test

To investigate the specific mechanism by which miR‐103‐3p regulates osteoblast activity, TargetScan, miRDB, and miRBase were used to predict the potential targets of miR‐103‐3p. The results showed that among the candidate target genes, *Mettl14* has a miR‐103‐3p binding site in its 3′ untranslated region (UTR) (Figure 3a). To test whether miR‐103‐3p directly targets *Mettl14*, dual‐luciferase reporters were constructed containing either a wild‐type (WT) *Mettl14* 3′UTR or a *Mettl14* 3′UTR mutant (MUT) sequence (Figure 3a). The luciferase reporter assay revealed that agomir‐103‐3p decreased WT *Mettl14* 3′UTR luciferase reporter activity, whereas antagomir‐103‐3p increased WT *Mettl14* 3′UTR luciferase reporter activity but not MUT *Mettl14* 3′UTR reporter activity (Figure 3b). By comparison, agomir‐NC or antagomir‐NC had no effect on luciferase activity after cotransfection with either the WT or MUT *Mettl14* 3′UTR (Figure 3b). To further confirm that miR‐103‐3p directly targets *Mettl14*, we constructed luciferase reporters that carried the *Mettl14* 3′UTR containing mutant sequences of the miR‐103‐3p binding site (agomir‐103‐3p‐Mut1 and agomir‐103‐3p‐Mut2) (Figure 3a). We found that agomir‐103‐3p, but not the two agomir‐103‐3p mutants, substantially inhibited the luciferase reporter activity of the WT *Mettl14* 3′UTR (Figure 3c), suggesting that these are likely true miR‐103‐3p targeting sites. RNA immunoprecipitation (RIP) assays with an Ago2 antibody followed by q‐PCR revealed a remarkable increase in recruitment of *Mettl14* mRNA to the miRNA complex in cells overexpressing miR‐103‐3p (Figure 3d). Further experiments showed that both *Mettl14* mRNA and protein levels were decreased in cells with agomir‐103‐3p but increased in cells with antagomir‐103‐3p (Figure 3e). To explore the potential role of miR‐103‐3p in m^6^A modification in osteoblasts, we examined the level of m^6^A in the total RNA and found that agomir‐103‐3p resulted in decreased m^6^A levels in osteoblasts and that antagomir‐103‐3p reversed the reduction in m^6^A levels (Figure 3f).

To examine whether miR‐103‐3p functionally targets *Mettl14* to regulate osteoblast activity, we knocked down *Mettl14* expression using siRNA (Figure 3g). We found that when we cotransfected cells with *Mettl14* siRNA‐NC and agomir‐103‐3p or antagomir‐103‐3p, there was no change in the effects of miR‐103‐3p on osteoblast proliferation (Figure S3a). However, when we cotransfected cells with siRNA‐*Mettl14* and agomir‐103‐3p or antagomir‐103‐3p, the effects of miR‐103‐3p on osteoblast proliferation were blocked (Figure S3b). Similar data were also obtained for osteoblast differentiation (Figure S3c and Figure 3h). To further confirm that miR‐103‐3p‐mediated regulation of osteoblast activity is *Mettl14* dependent, we used the WT *Mettl14* 3′UTR to block the binding of endogenous miR‐103‐3p to *Mettl14* (Figure 3i). The time‐dependent growth curve was shifted upward in cells transfected with the WT *Mettl14* 3′UTR compared to cells treated with the empty vector (Figure S3d). Similar data were also obtained for osteoblast differentiation (Figure 3j,k).

### 2.4 METTL14‐dependent m^6^A methylation regulates miR‐103‐3p processing by the microprocessor protein DGCR8

**FIGURE 4:**
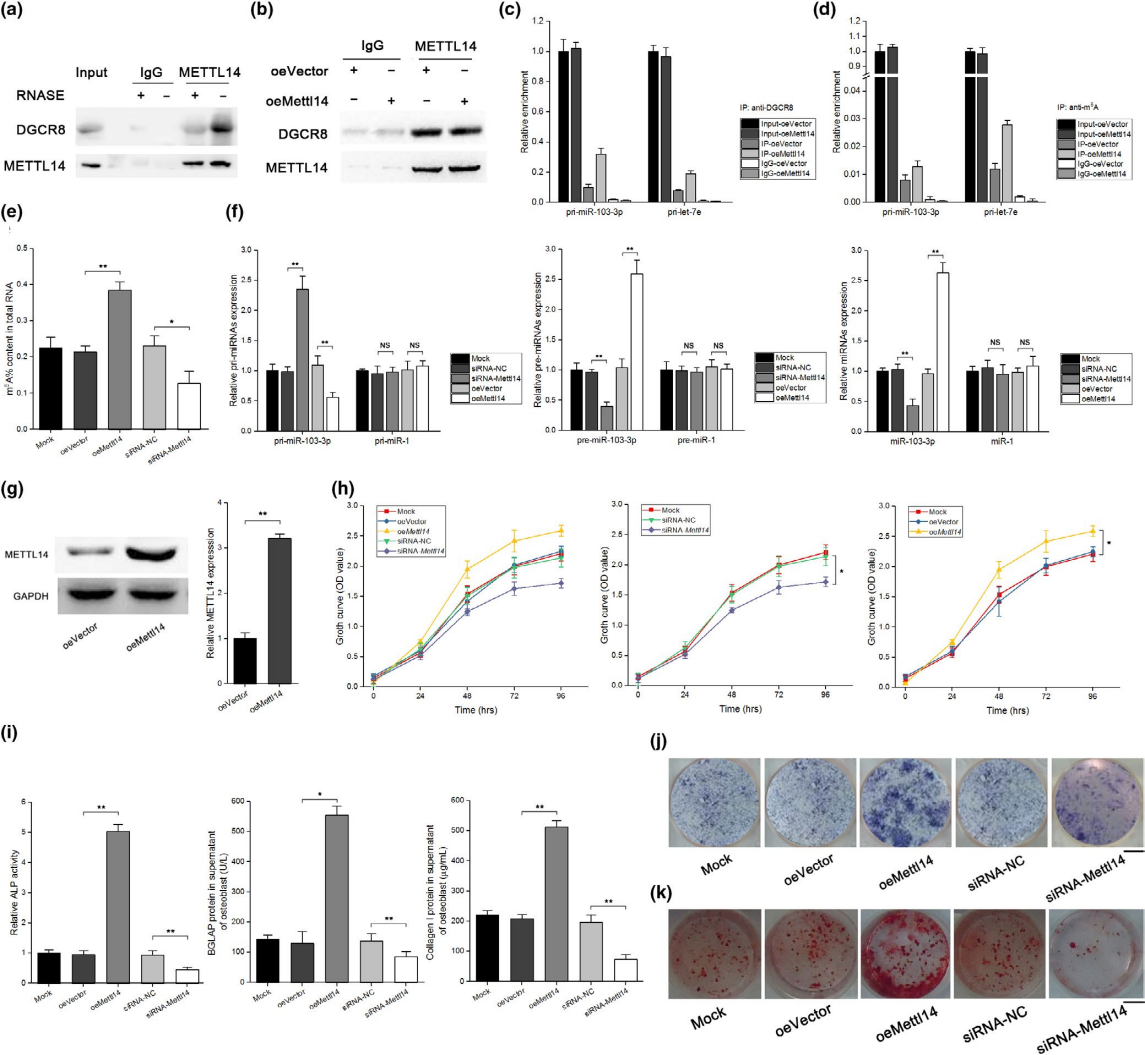
METTL14‐dependent m^6^A methylation regulates miR‐103‐3p processing by the microprocessor protein DGCR8 and modulates osteoblast activity in vitro. (a) Coimmunoprecipitation (IP) of the METTL14‐interacting protein DGCR8. Western blotting with anti‐DGCR8 and anti‐METTL14 antibodies and immunoglobulin G (IgG) antibody was used as a control for IP. (b) IP of DGCR8, METTL14, and associated RNA from control MC3 T3‐E1 cells or METTL14‐overexpressing MC3 T3‐E1 cells. The cells were UV‐cross‐linked before IP. Western blotting or immunoblotting was conducted using the antibodies described above. (c) Real‐time PCR analysis of pri‐miR‐103‐3p binding to DGCR8 in IP assay of DGCR8‐associated RNA from control and METTL14‐overexpressing MC3 T3‐E1 cells (*n* = 3). Pri‐let‐7e was used as a positive control. (d) Real‐time PCR analysis of the pri‐miR‐103‐3p m^6^A modification level determined by IP of m^6^A‐modified miRNA in control or METTL14‐overexpressing MC3 T3‐E1 cells (*n* = 3). Pri‐let‐7e was used as a positive control. (e) The effect of oe*Mettl14*, siRNA‐*Mettl14*, or the corresponding negative controls on the m^6^A content in total RNA (n = 3). (f) Real‐time PCR analysis of pri‐miR‐103‐3p (left), pre‐miR‐103‐3p (middle), and miR‐103‐3p (right) in METTL14 knockdown and overexpression MC3 T3‐E1 cells (*n* = 3). (g) Western blot analysis of the overexpression efficiency of *Mettl14* plasmid after treatment of primary mouse osteoblasts with a specific *Mettl14* plasmid (oe*Mettl14*) or an empty vector (*n* = 3). (h) WST‐8 assay of changes in primary mouse osteoblast growth at 24–96 h after treatment with oe*Mettl14*, siRNA‐*Mettl14* or the corresponding negative controls (*n* = 3). (i) ALP activity (left) and the amount of BALP protein (middle) and collagen I (right) in the supernatant of primary mouse osteoblasts after treatment with oe*Mettl14*, siRNA‐*Mettl14* or the corresponding negative controls (*n* = 3). (j) Representative images of ALP staining of primary mouse osteoblasts after treatment with oe*Mettl14*, siRNA‐*Mettl14*, or the corresponding negative controls (*n* = 3). Scale bar, 10 mm. (k) Alizarin red staining of calcium deposition in primary mouse osteoblasts after treatment with oe*Mettl14*, siRNA‐*Mettl14*, or the corresponding negative controls in osteogenic medium for 21 days. Scale bar, 10 mm. All data are the mean ± *SD*. *p < 0.05, **p < 0.01. One‐way ANOVA with a post hoc test was performed, and the significance of differences between two groups was determined with Student's *t* test

Previous studies have suggested that altered METTL3/m^6^A modification could participate in regulation of miRNAs in many biological processes (Alarcón et al., 2015; Han et al., 2019; Peng et al., 2019; Wang, Deng, et al., 2019; Wang, Ishfaq, et al., 2019; Yan et al., 2020; Zhang, Bai, et al., 2019). To identify whether altered METTL14/m^6^A modification is involved in regulating miR‐103‐3p in an m^6^A‐dependent pri‐miRNA‐processing manner, we first examined whether METTL14 was required for engagement of pri‐miRNAs by the microprocessor protein DGCR8. Immunoprecipitation assays showed that METTL14 coprecipitated with DGCR8. Ribonuclease treatment weakened this coprecipitation, suggesting that the interaction of METTL14 and DGCR8 might be mediated in part by RNAs (Figure 4a). Additionally, we found that methylated RNA bound by DGCR8 was increased in METTL14‐overexpressing (oeMETTL14) cells (Figure 4b). These findings indicated that METTL14 regulated pri‐miRNA processing by manipulating recognition and binding of pri‐miRNAs by DGCR8. When we immunoprecipitated DGCR8 from the vector and oeMETTL14 groups and used q‐PCR to examine pri‐miRNAs bound to DGCR8, we found that the level of pri‐miR‐103‐3p bound to DGCR8 was increased in oeMETTL14 cells (Figure 4c). Moreover, we discovered that the level of pri‐miR‐103‐3p modified by m^6^A was increased in oeMETTL14 cells when m^6^A was immunoprecipitated from RNAs of the vector and oeMETTL14 groups (Figure 4d). To further test the role of METTL14/m^6^A modification in miR‐103‐3p regulation, we first examined the level of m^6^A in the total RNA and found that oeMETTL14 resulted in increased m^6^A levels in osteoblasts and that siRNA‐METTL14 decreased the m^6^A level (Figure 4e). We then assessed the expression of pri‐miR‐103‐3p, pre‐miR‐103‐3p, and mature miR‐103‐3p in siRNA‐METTL14 or oeMETTL14 cells. Our data showed that the level of pri‐miR‐103‐3p was increased in the siRNA‐METTL14 group and decreased in the oeMETTL14 group. The levels of pre‐miR‐103‐3p and mature miR‐103‐3p were decreased in the siRNA‐METTL14 group and increased in the oeMETTL14 group (Figure 4f). We found a similar role of METTL14 in hFOB 1.19 cells (Figure S4a). Taken together, these results indicate that the presence of m^6^A enhanced the recognition of pri‐miR‐103‐3p by DGCR8 and the subsequent processing of pri‐miR‐103‐3p into mature miR‐103‐3p.

### 2.5 METTL14 promotes osteoblast proliferation, differentiation, and matrix mineralization

To explore the role of METTL14 in osteoblast activity, we transfected primary osteoblasts with siRNA‐METTL14 or the oeMETTL14 vector and then examined the efficiency of METTL14 overexpression and knockdown. METTL14 levels were upregulated by the oeMETTL14 vector and downregulated by siRNA‐METTL14 (Figures 3g and 4g). WST‐8 assays showed that the time‐dependent growth curve was shifted downward in siRNA‐METTL14 cells, whereas the growth curve was shifted upward in oeMETTL14 cells compared to cells treated with the corresponding negative controls (siRNA‐NC and oeVector) (Figure 4h). Additionally, the *Alp*, *Bglap*, and *Col1α1* levels were downregulated by siRNA‐METTL14 and upregulated by oeMETTL14 compared to the corresponding control treatments (Figure S4b). ALP activity and the amount of BGLAP protein and collagen I in the supernatant of primary mouse osteoblasts were lower in the siRNA‐METTL14 treatment group and higher in the oeMETTL14 treatment group than in the siRNA‐NC and oeVector treatment groups, respectively (Figure 4i). We found similar effects of METTL14 in hFOB 1.19 cells (Figure S4c‐e). In addition, ALP staining assays showed that siRNA‐METTL14 weakened ALP staining in primary mouse osteoblasts, whereas oeMETTL14 enhanced ALP staining in the cells (Figure 4j). Moreover, we performed an Alizarin red staining assay and found that siRNA‐METTL14 decreased mineral deposition, whereas oeMETTL14 increased mineral deposition compared the corresponding control treatments (Figure 4k).

### 2.6 miR‐103‐3p inhibits bone formation in vivo

**FIGURE 5:**
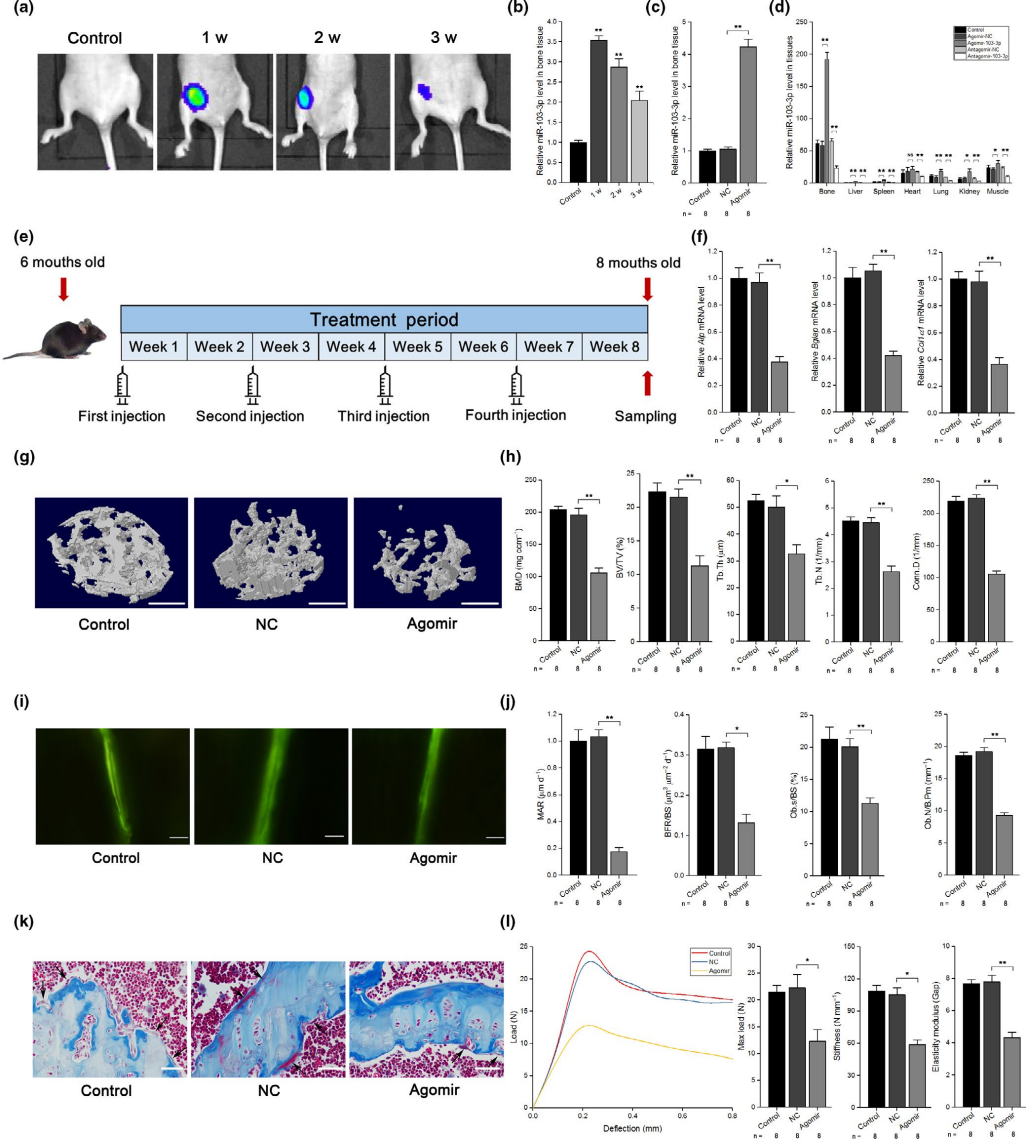
miR‐103‐3p inhibits bone formation in vivo. (a) Live animal fluorescence imaging analysis of GFP expression at 1–3 weeks postinjection (*n* = 3). (b) Real‐time PCR analysis of miR‐103‐3p levels in bone specimens from mice at 1–3 weeks after treatment with agomir‐103‐3p (*n* = 3). (c) Real‐time PCR analysis of miR‐103‐3p levels in bone specimens from mice after treatment with agomir‐103‐3p or its negative control. (d) Real‐time PCR analysis of miR‐103‐3p levels in different tissues after a single injection of agomir‐103‐3p, antagomir‐103‐3p, or the corresponding negative controls (*n* = 3). The relative miRNA levels in the liver tissue were normalized to the mean value of the control group. (e) Schematic diagram illustrating the experimental design. (f) Real‐time PCR analysis of *Alp* (left), *Bglap* (middle), and *Col1α1* (right) mRNA levels in bone specimens from mice after treatment with agomir‐103‐3p or its negative control. (g) Representative images showing the three‐dimensional trabecular architecture in distal femurs determined by microCT reconstruction. Scale bars, 1 mm. (h) MicroCT measurements of BMD, BV/TV, Tb.Th, Tb.N, and Conn.D in the distal femurs of mice after treatment with agomir‐103‐3p or its negative control. (i) Representative images of new bone formation assessed by double calcein labeling. Scale bars, 10 μm. (j) Double calcein labeling analysis of dynamic bone histomorphometric parameters (MAR, BFR/BS, Ob.s/BS, and Ob.N/B.Pm) in the distal femur of mice after treatment with agomir‐103‐3p or its negative control. (k) Masson's trichrome staining analysis of osteoid formation in the distal femur of mice after treatment with agomir‐103‐3p or its negative control (*n* = 8). Scale bars, 10 μm. Arrows indicate osteoids. (l) Three‐point bending test analysis of mouse femur biomechanical properties after treatment with agomir‐103‐3p or its negative control. The *n* value for each group is indicated at the bottom of each bar in the graphs. All data are presented as the mean ± *SD*. *p < 0.05, **p < 0.01. One‐way ANOVA with a post hoc test was performed, and the significance of differences between two groups was determined with Student's *t* test

To determine the effects of miR‐103‐3p on bone formation, we constructed an adeno‐associated virus that expressed green fluorescent protein (GFP) and agomir‐103‐3p or antagomir‐103‐3p as an in vivo delivery system. To investigate the effectiveness of the delivery system in vivo, we injected the delivery system via periosteal injection into the marrow cavity of the femur with an in‐house pressurized injector and tracked GFP expression in live mice with a whole‐animal fluorescence imaging system (Li et al., 2015; Wang, Deng, et al., 2019; Xu et al., 2018) at 1, 2, and 3 weeks postinjection (Figure 5a). No fluorescence was detected in the control group, and the GFP fluorescence signals in the right femur lasted up to 3 weeks postinjection (Figure 5a). To further confirm the expression of miR‐103‐3p in the bone tissue of mice, q‐PCR analysis was performed and showed that the level of miR‐103‐3p was increased and that this increase lasted for 3 weeks after a single periosteal injection; in addition, the variation in miR‐103‐3p levels was greater in bone than in other tissues (Figure 5b‐d and Figure S5a).

To further test the effects of miR‐103‐3p on bone formation in vivo, we performed pulsed periosteal injections of agomir‐103‐3p (agomir) or agomir‐NC (NC) with the delivery system in mice at 6 months (Figure 5e). We chose the *Alp*, *Bglap*, and *Col1α1* levels as indicators of osteoblast activity in vivo, as described previously (Wang et al., 2013). q‐PCR analysis showed that the *Alp*, *Bglap*, and *Col1α1* levels were significantly lower than those in the NC group, and there were no differences between the control and NC groups (Figure 5f).

To analyze the bone mass and trabecular architecture of the mice, micro‐computed tomography (microCT) scanning of the distal femurs was performed in each group. The cortical bone thickness (Ct.Th) was lower in the femurs of agomir mice compared to NC mice (Figure S5b‐c). In addition, seven parameters were selected to investigate bone mass and the trabecular architecture: bone mineral density (BMD), the bone volume‐to‐total volume ratio (BV/TV), trabecular thickness (Tb.Th), trabecular number (Tb.N), connectivity density (Conn.D), trabecular separation (Tb.Sp), and the structural model index (SMI). The trabecular bone mass was significantly reduced (lower in both BMD and BV/TV), and the trabecular architecture was markedly impaired (lower Tb.Th, Tb.N, and Conn.D and higher Tb.Sp and SMI) in agomir mice compared to NC mice (Figure 5g,h and Figure S5d).

Six parameters were chosen for dynamic bone histomorphometric analysis via calcein double labeling: the mineral apposition rate (MAR), bone formation rate (BFR), ratio of osteoblast surface to bone surface (Ob.S/BS), osteoblast number per bone perimeter (Ob.N/B.Pm), osteoclast number per bone perimeter (Oc.N/B.Pm), and ratio of osteoclast surface to bone surface (Oc.S/BS). The values of bone formation‐related parameters (MAR, BFR, Ob.S/BS, and Ob.N/B.Pm) were lower in agomir mice than in NC mice, and the values of bone resorption‐related parameters (Oc.N/B.Pm and Oc.S/BS) were unchanged (Figure 5i,j and Figure S5e). Through tartrate‐resistant acid phosphatase (TRAP) staining, we also found that agomir‐103‐3p had no effect on osteoclast activity in vivo (Figure S5f‐h).

Masson's trichrome staining showed less osteoid staining in distal femurs from agomir mice than in those from NC mice (Figure 5k). To identify the effects of miR‐103‐3p on the mechanical properties of the femurs, we performed a three‐point bend test and found that the maximum load, stiffness, and elasticity modulus were decreased in the agomir group compared with the NC group (Figure 5i).

Further experiments showed that both *Mettl14* mRNA and protein levels were decreased in agomir mice but increased in antagomir mice (Figure S5i). Furthermore, agomir‐103‐3p resulted in decreased m^6^A levels, whereas antagomir‐103‐3p increased the level of m^6^A in mouse femurs (Figure S5j).

### 2.7 Inhibition of miR‐103‐3p promotes bone formation in OVX mice.

**FIGURE 6:**
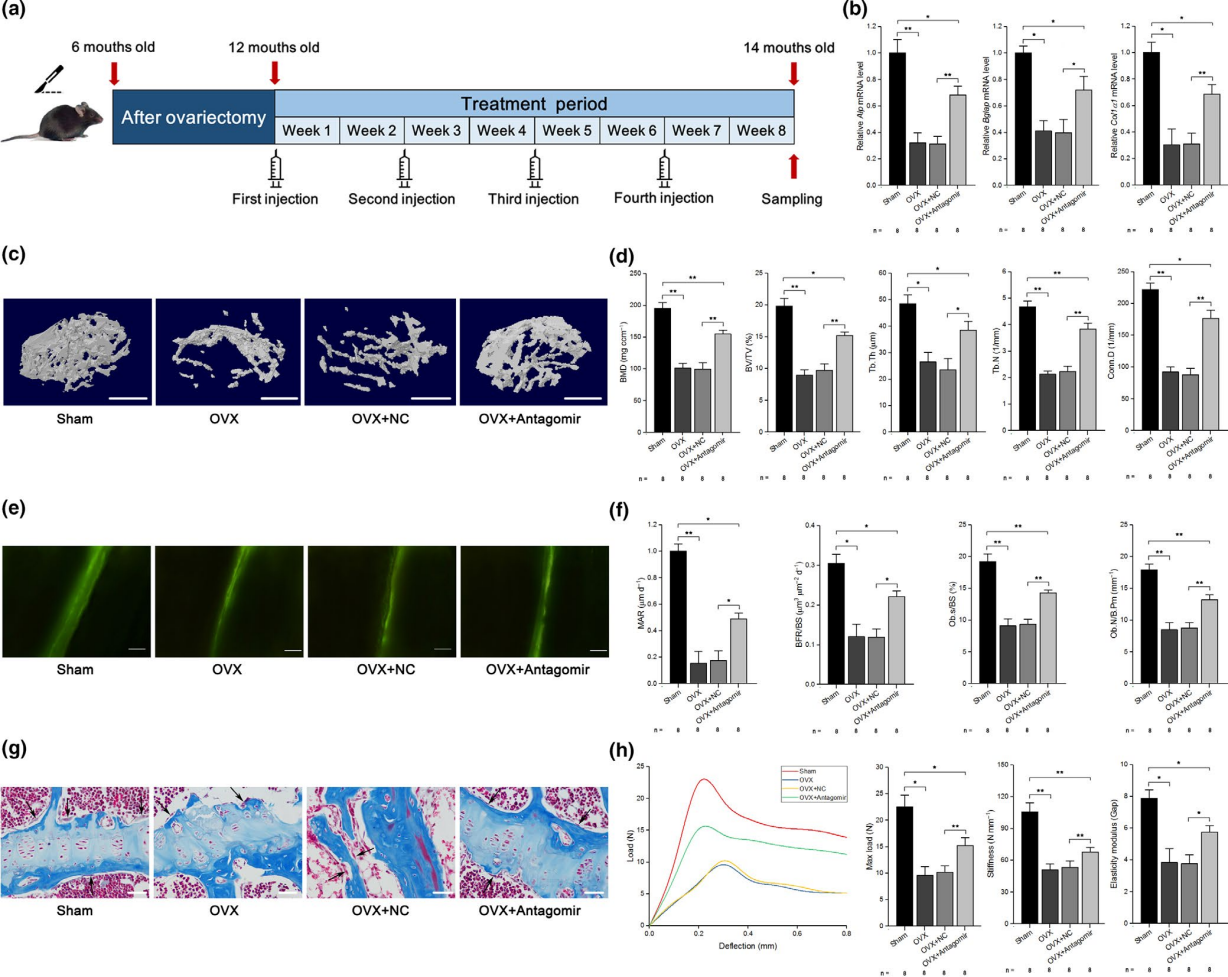
Therapeutic inhibition of miR‐103‐3p counteracts the decrease in bone formation in mice with OVX‐induced osteoporosis. (a) Schematic diagram illustrating the experimental design. (b) Real‐time PCR analysis of *Alp* (left), *Bglap* (middle), and *Col1α1* (right) mRNA levels in bone specimens from mice after treatment with antagomir‐103‐3p or its negative control. (c) Representative images showing three‐dimensional trabecular architecture determined by microCT reconstruction in the distal femurs. Scale bars, 1 mm. (d) MicroCT measurement for BMD, BV/TV, Tb.Th, Tb.N, and Conn.D in the distal femurs of mice after treatment with antagomiR‐103‐3p or its negative control. (e) Representative images of new bone formation assessed by double calcein labeling. Scale bars, 10 μm. (f) Double calcein labeling analysis of dynamic bone histomorphometric parameters (MAR, BFR/BS, Ob.s/BS, and Ob.N/B.Pm) in the distal femurs of mice after treatment with antagomiR‐103‐3p or its negative control. (g) Masson's trichrome staining analysis of osteoid formation in the distal femurs of mice after treatment with antagomiR‐103‐3p or its negative control (*n* = 8). Scale bars, 10 μm. Arrows indicate osteoids. (h) Three‐point bending test analysis of mouse femur biomechanical properties after treatment with antagomiR‐103‐3p or its negative control. The *n* value for each group is indicated at the bottom of each bar in the graphs. All data are presented as the mean ± *SD*. *p < 0.05, **p < 0.01. One‐way ANOVA with a post hoc test was performed, and the significance of differences between two groups was determined with Student's *t* test

To test the efficiency of the delivery system with antagomir‐103‐3p in vivo, q‐PCR analysis was performed and showed that the level of miR‐103‐3p was decreased and that this effect could last for 3 weeks postinjection; the variation in miR‐103‐3p levels was greater in bone than in other tissues (Figures S5a and S6a,b). To examine the therapeutic effects of antagomir‐103‐3p on bone formation in OVX‐induced osteoporotic mice, we performed pulsed injections of antagomir‐103‐3p (OVX+antagomir) or antagomir‐NC (OVX+NC) with the delivery system in estrogen‐depleted mice at 6 months after OVX (Figure 6a).

We found that the *Alp*, *Bglap*, and *Col1α1* levels were significantly lower in OVX mice or OVX mice treated with a negative control antagomir (OVX+NC) than in OVX+antagomir mice (Figure 6b). We also found that the cortical bone thickness was lower in the OVX and OVX+NC mice compared to sham mice and higher in the OVX+antagomir group compared to the OVX+NC group (Figure S6c,d). Additionally, trabecular bone mass was notably reduced (lower BMD and BV/TV) and that the trabecular architecture was significantly impaired (lower Tb.Th, Tb.N, and Conn.D and higher Tb.Sp and SMI) in OVX and OVX+NC mice compared to sham mice, whereas the trabecular bone mass was substantially increased, and the trabecular architecture was markedly improved in the OVX+antagomir group compared to the OVX+NC group (Figure 6c,d and Figure S6e). Furthermore, dynamic bone histomorphometric analysis showed that the values of bone formation‐related parameters (MAR, BFR, Ob.S/BS, and Ob.N/B.Pm) were lower and those of bone resorption‐related parameters (Oc.N/B.Pm and Oc.S/BS) were higher in OVX and OVX+NC mice than in sham mice, whereas bone formation‐related parameters were increased and bone resorption‐related parameters were not altered in OVX+antagomir mice compared to OVX+NC mice (Figure 6e,f and Figure S6f). Using TRAP staining, we also found that antagomir‐103‐3p had no effect on osteoclast activity in vivo (Figure S5f‐h). In addition, Masson's trichrome staining showed less osteoid staining in the distal femur in OVX and OVX+NC mice than in sham mice, whereas there was more osteoid staining in OVX+antagomir mice than in OVX+NC mice (Figure 6g). Next, we performed a three‐point bend test to test for changes in the mechanical properties of the femurs and identified that the maximum load, stiffness, and elasticity modulus were decreased in OVX and OVX+NC mice compared to sham mice, whereas these three biomechanical parameters were increased in OVX+antagomir mice compared to OVX+NC mice (Figure 6h). Our data also showed that both *Mettl14* mRNA and protein levels were decreased in OVX and OVX+NC mice compared to sham mice, whereas both *Mettl14* mRNA and protein levels were increased in OVX+antagomir mice compared to OVX+NC mice (Figure S6 g). We found a similar role of antagomir‐103‐3p in regulating m^6^A levels in mice with OVX‐induced osteoporosis (Figure S6h).

### 2.8 Correlations between the miR‐103‐3p/METTL14/m^6^A axis and bone formation capacity

To clarify the relationship between the miR‐103‐3p/METTL14/m^6^a axis and bone formation capacity, we examined both *Mettl14* mRNA and protein levels and m^6^A levels in bone specimens collected from 24 elderly female patients with low‐energy fractures (Figure 1a and Tables S1 and S2). Our results showed that the METTL14 protein levels, *METTL14* mRNA levels, and m^6^A content in total RNA were lower in osteoporosis patients (T ≤ −2.5) than in control patients (T ≥ −2.5) (Figure S7a). Then, we found that *METTL14* mRNA levels were positively correlated with the m^6^A content in total RNA (Figure S7b). We also found that miR‐103‐3p levels were negatively correlated with *METTL14* mRNA levels and the m^6^A content in total RNA from these human samples (Figure S7c). In addition, we showed that both the *METTL14* mRNA levels and m^6^A content in total RNA were positively correlated with *ALP*, *BGLAP*, and *COL1α1* levels in human bone tissue (Figure S7d,e).

We further confirmed the miR‐103‐3p/METTL14/m^6^a axis in bone tissues from OVX mice and found that the METTL14 protein levels, *Mettl14* mRNA levels, and m^6^A content in total RNA were lower in OVX mice than in sham mice (Figure S7f). Consistently, we found that *Mettl14* mRNA levels were positively correlated with the m^6^A content in total RNA (Figure S7 g). We also found that miR‐103‐3p levels were negatively correlated with *Mettl14* mRNA levels and the m^6^A content in total RNA from the mouse bone samples (Figure S7 h). In addition, we determined that both *Mettl14* mRNA levels and the m^6^A content in total RNA were positively correlated with *Alp*, *Bglap*, and *Col1α1* levels in the bone tissue of the mice (Figure S7i,j).

Further experiments showed that the *Alp*, *Bglap*, and *Col1α1* levels gradually increased and the level of miR‐103‐3p decreased with osteoblast differentiation (Figure S7 k). Moreover, the METTL14 protein levels, *Mettl14* mRNA levels, and m^6^A content in total RNA increased with osteoblast differentiation (Figure S7 l).

## 3 DISCUSSION

In the present study, we found that the miR‐103‐3p level was negatively correlated with bone formation in bone specimens from elderly women with fractures as well as in OVX mice. Then, we found that miR‐103‐3p directly targeted *Mettl14* to functionally inhibit osteoblast activity and that METTL14 provided negative feedback to regulate miR‐103‐3p processing by DGCR8 and promote osteoblast activity. Moreover, we presented in vivo evidence demonstrating that miR‐103‐3p inhibited bone formation under physiological conditions and that inhibition of miR‐103‐3p promoted bone formation in OVX mice. In addition, we confirmed that *METTL14* was negatively correlated with miR‐103‐3p but positively correlated with bone formation in bone specimens from women with fractures and OVX mice. As summarized in Figure S7 m, we elucidated the miR‐103‐3p/METTL14/m^6^a signaling axis in osteoblasts and highlighted the critical roles of this signaling axis in reduced bone mass in postmenopausal osteoporosis.

A series of miRNAs have been verified to regulate osteoblast function and osteoblastic bone formation (Inose et al., 2009; Wang et al., 2013; Xu et al., 2018). We found a close association between elevated miR‐103‐3p and reduced bone formation, evidenced by the negative correlation between the miR‐103‐3p level and *ALP*, *BGLAP*, and *COL1α1* mRNA levels in bone specimens from elderly women with fractures and in OVX mice and primary mouse osteoblasts. These results prompted us to investigate whether miR‐103‐3p plays a role in osteoblast activity. miR‐103‐3p has been reported to be adversely correlated with osteoblast differentiation and bone formation in response to mechanical stimulation (Zuo et al., 2015). Our previous study showed that miR‐103‐3p inhibits osteoblast proliferation under simulated microgravity conditions, a form of mechanical unloading (Sun, Cao, Hu, et al., 2015; Sun, Cao, Zhang, et al., 2015). Consistently, we observed that miR‐103‐3p inhibited osteoblast activity in mouse primary osteoblasts and hFOB 1.19 cells. More importantly, we found that miR‐103‐3p had no effect on osteoclast activity. Therefore, these data suggest that the aberrantly elevated miR‐103‐3p in bone specimens may contribute to a reduction in osteoblast activity and suppression of bone formation.

Some mechanisms by which miR‐103‐3p regulates gene expression are already known (Favereaux et al., 2011; Sun, Cao, Zhang, et al., 2015; Trajkovski et al., 2011). Specifically, miR‐103‐3p directly targets caveolin‐1 to regulate insulin sensitivity (Trajkovski et al., 2011). Favereaux et al. showed that miR‐103‐3p is directly involved in chronic pain via targeting of Cav1.2 (Favereaux et al., 2011). In the present study, we predicted the potential targets of miR‐103‐3p by using miRNA target prediction software to investigate how miR‐103‐3p regulates osteoblast activity. Notably, the 3’UTR of *Mettl14* possesses a 7‐nt perfect match site for the miR‐103‐3p seed region predicted by prediction tools. Moreover, we confirmed that *Mettl14* is a direct target of miR‐103‐3p in MC3 T3‐E1 mouse preosteoblasts, as indicated by the luciferase assay and RIP analysis. miR‐103‐3p negatively regulated METTL14 expression and the m^6^A level in total RNA from primary mouse osteoblasts. Further functional studies verified that miR‐103‐3p inhibits osteoblast activity by directly targeting *Mettl14* in osteoblasts. Therefore, our present study found for the first time that *Mettl14* is a new target of miR‐103‐3p in osteoblasts.

Previous studies have shown that altered m^6^A levels are involved in regulation of miRNAs (Alarcón et al., 2015; Han et al., 2019; Ma et al., 2017; Peng et al., 2019; Wang, Deng, et al., 2019; Yan et al., 2020; Zhang, Bai, et al., 2019). Alarcón and colleagues showed that m^6^A modification promotes the recognition of pri‐miRNA sequences and reported that DGCR8 is involved in an initiation event in miRNA biogenesis during METTL3‐dependent m^6^A methylation (Alarcón et al., 2015). Other researchers have demonstrated that upregulation of METTL3/m^6^A modification promotes pri‐miR‐25 (Zhang, Bai, et al., 2019), pri‐miR‐221/222 (Han et al., 2019), pri‐miR‐143‐3p (Wang, Deng, et al., 2019), and pri‐miR‐1246 (Peng et al., 2019) maturation (decreasing the expression of pri‐miRNA but increasing the expression of pre‐miRNA and miRNA). Recently, Ma et al. reported that METTL14, another important m^6^A methyltransferase regulator, positively modulates the pri‐miR‐126 process in a DGCR8‐dependent manner (Ma et al., 2017). In the present study, we showed that METTL14‐dependent m^6^A methylation regulated miR‐103‐3p processing by the microprocessor protein DGCR8. However, some reports have demonstrated that METTL3/m^6^A suppresses the expression of pre‐miR‐320 and miR‐320 (Yan et al., 2020). This variation in results may be due to the use of different species and cell sources or different disease models.

Accumulating evidence has shown that m^6^A modification not only modulates nearly all aspects of RNA metabolism, such as splicing, structure, stability, translation, and export, but is also involved in many human diseases, including major depressive disorder, obesity‐related traits, type 2 diabetes mellitus, and cancers (Wang, & He, 2014; Zhao, & He, 2015; Zhao et al., 2017). Recently, several studies have focused on the involvement of m^6^A modification in bone metabolism (Guo et al., 2011; Li et al., 2019; Sachse et al., 2018; Shen et al., 2018; Tian et al., 2019; Wu et al., 2018; Yao et al., 2019; Yu et al., 2020; Zhang, Riddle, et al., 2019; Zhang et al., 2020). Wu and colleagues demonstrated that METTL3‐mediated m^6^A modification regulated the fate of bone marrow mesenchymal stem cells (Wu et al., 2018). Other studies have shown that the m^6^A demethylase FTO is essential for normal bone growth and functions to protect osteoblasts from genotoxic damage (Zhang, Riddle, et al., 2019). However, the biological significance of the m^6^A methyltransferase METTL14 in bone formation or osteoporosis has not been confirmed. In this study, we showed for the first time that METTL14 promotes osteoblast proliferation, differentiation, and matrix mineralization.

As important therapeutic targets, miRNAs are being studied in preclinical and clinical studies (Bouchie, 2013; Janssen et al., 2013). Recently, several studies have tested the efficacy of miRNA‐modulating compounds in combating osteoporosis. Wang and colleagues demonstrated that therapeutic silencing of miR‐214 could increase bone formation and counteract osteoporosis (Wang et al., 2013). Another study showed that inhibition of miR‐31a‐5p prevented bone loss in aged rats (Xu et al., 2018). Our previous studies revealed that blocking the expression of miRNA‐132‐3p reverses disuse osteopenia in mice (Hu et al., 2020). We also showed that delivery of miR‐33‐5p partially rescued bone loss in hindlimb‐unloaded mice (Wang et al., 2018). In the present study, we showed that mature miR‐103‐3p is evolutionarily conserved among several species and highly expressed in bone tissue. Moreover, we demonstrated that miR‐103‐3p inhibited osteoblast activity and bone formation by targeting *Mettl14* in vivo under physiological conditions. More importantly, the therapeutic inhibition of miR‐103‐3p partially counteracted bone loss in OVX mice. In addition, miR‐103‐3p had no effect on osteoclast activity in vitro and in vivo. These results demonstrate that therapeutic silencing of miR‐103‐3p may promote bone formation and partially rescue bone loss in postmenopausal osteoporosis by exerting an anabolic effect on osteoblast activity. To reveal the relationship between the miR‐103‐3p/METTL14/m^6^a axis and bone formation capacity, we measured METTL14 expression levels in bone specimens and found that *METTL14* was negatively correlated with miR‐103‐3p but positively correlated with bone formation in bone specimens from elderly women with fractures and OVX mice.

It should be noted that our study has some limitations. In the present study, we observed a regulatory effect of miR‐103‐3p/METTL14/m^6^a on osteoblast activity but did not investigate the effect and mechanism of METTL14 in vivo. We are working to acquire a sufficient number of osteoblast‐specific miR‐103‐3p and METTL14 knock‐out and knock‐in mice to further verify the existence of the miR‐103‐3p/METTL14/m^6^a axis in vivo and test the mechanism of METTL14 in bone formation. In addition, unfortunately, due to the experimental material constraints, we did not get or synthesize the targeted delivery systems in our laboratory, and we would further test the effects of miR‐103‐3p modulators on osteoblast in vivo in a future study.

In summary, our results illustrate that miR‐103‐3p plays critical roles in postmenopausal osteoporosis by inhibiting osteoblast activity and bone formation and reveal a previously unrecognized signaling axis involving miR‐103‐3p/METTL14/m^6^A in osteoblasts. Moreover, our work suggests that this signaling axis may be a potential target for ameliorating postmenopausal osteoporosis.

## 4 EXPERIMENTAL PROCEDURES

The detailed procedures were provided in the Appendix S1.

## CONFLICT OF INTEREST

The authors declare no competing financial interests.

## AUTHORS' CONTRIBUTIONS

Z.S., H.W., Y.W., and G.Y. performed the majority of the experiments, analyzed data, and prepared the manuscript. Z.S., X.Y., H.J., Q.W., and B.Y. collected human bone samples. Z.S. and Z.H. helped with in vivo treatment. X.Y., H.J., Q.W., F.S., and Z.S. assisted with in vitro experiments. Z.S. and B.Y. maintained the mice. Z.S. helped with microdissection analysis. Z.S. and B.Y. helped with data analysis. S.Z., T.G., and J.Z. supervised the project.

## Supporting information

Appendix S1

## Data Availability

We provided part of raw data and ethics approval statements in the Appendix S1. If necessary, we provided all the original data.
