# Supplementary material for: MiR‐103‐3p targets the m6A methyltransferase METTL14 to inhibit osteoblastic bone formation
Source: Aging Cell. 2021 Jan 13;20(2):e13298. doi: 10.1111/acel.13298 (PMC7884043; doi:10.1111/acel.13298)
Supplement: Supplementary file 1 — Appendix S1 [file ACEL-20-e13298-s001.docx]

**MiR-103-3p targets the m^6^A methyltransferase METTL14 to inhibit osteoblastic bone formation**

Running title: miR-103-3p inhibits bone formation

Zhongyang Sun^1,2a^, Han Wang^3a^, Yuxiang Wang^1a^, Guodong Yuan^4a^, Xin Yu^1^, Hui Jiang^1^, Qi Wu^1^, Binkui Yang^5^, Zebing Hu^6^, Fei Shi^6^, Xinsheng Cao^6^, Shu Zhang^6*^, Ting Guo^1*^, Jianning Zhao^1*^

^1^Department of Orthopedics, Affiliated Jinling Hospital, Medical School of Nanjing University, Nanjing, China 210093.

^2^Department of Orthopedics, Air Force Hospital of Eastern Theater, Anhui Medical University, Nanjing, China 210002.

^3^Department of Orthopedics, Air Force Medical Center, PLA, Beijing, China 100142.

^4^Department of Orthopedics, Medical School of Southeast University, Nanjing, China 210093.

^5^Hangzhou Special Sanatorium Center of the PLA Air Force, Nanjing, China 210093.

^6^The Key Laboratory of Aerospace Medicine, Ministry of Education, Air Force Medical University, Xi'an, China 710032.

^a^These authors contributed equally to this work

^*^To whom correspondence should be addressed:

Shu Zhang: shuzhang89@hotmail.com; Tel. (86) 29-84711231; Fax. (86) 29-84711215.

Ting Guo: guotingli@vip.sina.com; Tel. (86) 25-80865210; Fax. (86) 25-80865210.

Jianning Zhao: zhaojianning.0207@163.com; Tel. (86) 25-80860015; Fax. (86) 25-80860015.

**Experimental procedures**

**4.1 Human bone tissue preparation**

The bone tissues of 10 female osteoporotic patients and 14 female nonosteoporotic patients were collected from two clinical settings (Affiliated Jinling Hospital, Medical School of Nanjing University, and the Air Force Hospital of the Eastern Theater, Anhui Medical University; Table S1 and Table S2). Female patients between 65 and 90 years of age were recruited. Elderly postmenopausal women with proximal femur or femoral neck fracture treated with femoral head replacement or total hip replacement were included (inclusion criteria). Patients who were currently treated with medications that can alter bone turnover were excluded. Moreover, patients with diabetes, malignancy, coronary heart disease or other severe diseases in the previous 5 years were excluded from the present study (exclusion criteria). The bone biopsies were cancellous bone taken from proximal femur of the patients with femoral head replacement or total hip replacement. It is notable that the fat and connective tissues need to be stripped and bone marrow needs to be washed out before freezing (Wang et al., 2013; Li et al., 2016). It took within 10 minutes from sampling to freezing. The patients were divided into T>−2.5 and T≤−2.5 groups according to the T-score for bone mineral density in the spine determined by dual-energy X-ray absorptiometry evaluation. All clinical procedures were approved by the Committees of Clinical Ethics of the Affiliated Jinling Hospital, Medical School of Nanjing University (Nanjing, China; Reference number: 20180101005), and the Air Force Hospital of the Eastern Theater, Anhui Medical University (Nanjing, China; Reference number: 20180305013) and conformed to the principles of the Helsinki Declaration. We obtained informed consent from all the participants.

**4.2 OVX-induced osteoporotic mouse model**

The female C57BL/6J mice used in our study were obtained from the Laboratory Animal Center of Jiangsu Province. The animals were housed in an animal room under standard temperature (25°C), humidity (55-60%), and light-dark cycle (12 h/12 h) condi­tions. Animals were given free access to a standard diet and tap water. The mice were ovariectomized or sham-operated at 6 months of age. At 6 months after surgery (12 months of age), femurs from ovariectomized or sham-operated mice were sampled and tested (Wang et al., 2013; Li et al., 2016). All the experimental procedures were approved by the Committees of Animal Ethics and Experimental Safety of the Affiliated Jinling Hospital, Medical School of Nanjing University (Nanjing, China; Reference number: 20180101006) and the Air Force Hospital of the Eastern Theater, Anhui Medical University (Nanjing, China; Reference number: 20180305014), in accordance with the National Institutes of Health Guide for the Care and Use of Laboratory Animals.

**4.3 Cell culture and transfection**

Primary osteoblasts were derived from 24-h postnatal female C57BL/6J mouse calvarias by sequential digestion in a 0.1% collagenase I (Sigma-Aldrich, Missouri, USA) and 0.25% trypsin (Sigma-Aldrich, Missouri, USA) mixture (Li et al., 2016). Cells from the third and subsequent digests were cultured in α-modified Eagle’s medium (HyClone, Utah, USA) with 10% fetal bovine serum (HyClone, Utah, USA) and 1% penicillin and streptomycin (HyClone, Utah, USA). The cells were subcultured every 3 days and used from passages 3 to 5. Then, the cells were confirmed to be osteoblasts using a phenotype characterized by RUNX2 and OSX expression and the capacity to form mineralized bone nodules (Figure S1f-g). The hFOB 1.19 cell line and the MC3T3-E1 cell line were maintained in α-modified Eagle’s medium (HyClone, Utah, USA) with 10% fetal bovine serum (HyClone, Utah, USA) and 1% penicillin and streptomycin (HyClone, Utah, USA) (Wang et al., 2013). The cell lines were not used beyond passage 10. These osteoblasts were cultured in medium with 10 nM dexamethasone (Sigma-Aldrich, Missouri, USA), 50 μg/ml ascorbic acid (Sigma-Aldrich, Missouri, USA) and 5 mM β-glycerophosphate (Sigma-Aldrich, Missouri, USA). After 3 weeks, the cells were subjected to Alizarin red staining (Sigma-Aldrich, Missouri, USA).

To obtain mature primary mouse osteoclasts, bone marrow cells were isolated from the long bones of female C57BL/6J mice and cultured in α-modified Eagle’s medium (HyClone, Utah, USA) with 10% fetal bovine serum (HyClone, Utah, USA) and 1% penicillin and streptomycin (HyClone, Utah, USA). Then, the cells were cultured in α-modified Eagle’s medium for another 2 days with 25 ng/ml M-CSF (PeproTech, New Jersey, USA) and for an additional 6 days in the same medium with 25 ng/ml mouse M-CSF and 5 ng/ml mouse RANKL (PeproTech, New Jersey, USA).

For transfection with miRNA or siRNA oligos, the medium was mixed with Lipofectamine 2000 (Invitrogen, California, USA), which was used according to the manufacturer's protocol. Cells were transfected with miRNA oligos (agomir-NC, agomir-103-3p, agomir-103-3p-Mut1, agomir-103-3p-Mut2, antagomir-NC and antagomir-103-3p) at a concentration of 300 μM. Cells were transfected with siRNA oligos (siRNA-NC and siRNA-*Mettl14*) at a concentration of 500 μM. Cells were transfected with other oligos (WT *Mettl14* 3'UTR and Mut *Mettl14* 3'UTR) at a concentration of 300 μM. The oligonucleotide sequences used in this study were previously described by Sun *et al.* (Sun, Cao, Hu et al., 2015; Sun, Cao, Zhang et al., 2015; Ma, et al., 2017) and are listed in Table S3.

**4.4 miRNA and mRNA expression assays**

Total RNA was extracted from collected bone specimens and cells with TRIzol reagent (Invitrogen, California, USA) (Wang et al., 2013). The quality of the isolated RNA was tested by examining absorbance at 260 and 280 nm with a NanoDrop Spectrophotometer (Thermo, Massachusetts, USA).

For miRNA, cDNA was synthesized using a miRNA First Strand Synthesis Kit (Agilent Technologies, Beijing, China). Amplification was carried out at 95°C for 2 min, followed by 40 cycles of 95°C for 10 s and 60°C for 40 s. For mRNA, cDNA was synthesized using a PrimeScript RT Kit (Takara, Tokyo, Japan). The amplification was performed at 95°C for 45 s, followed by 40 cycles of 58°C for 45 s and 72°C for 60 s (Sun, Cao, Hu et al., 2015; Sun, Cao, Zhang et al., 2015; Sun et al., 2019). The primer sequences used for real-time PCR were previously described by Sun *et al.* (Wang et al., 2013; Sun, Cao, Zhang et al., 2015; Li et al., 2016; Zhang et al., 2019; Han et al., 2019; Wang, Deng et al.,2019) and are listed in Table S4.

**4.5 Cell proliferation assay**

Cell proliferation was tested with a WST-8 (4-{3-(2-methoxy-4-nitrophenyl)-2-(4-nitrophenyl)-2H-5-tetrazolio}-1,3-benzene disulfonate sodium salt; Cell Counting Kit-8, Dojindo, Tokyo, Japan) assay according to the manufacturer’s protocol (Sun, Cao, Hu et al., 2015). The absorbance of the reaction solution at 450 nm was measured with a microplate reader (Molecular Devices, California, USA). We also performed 5-ethynyl-2'-deoxyuridine (EdU) labeling with an EdU Detection Kit (RiboBio, Guangzhou, China) to test cell proliferation. The cells were stained with a reaction cocktail (RiboBio, Guangzhou, China) and Hoechst 33342 (Sigma-Aldrich, Missouri, USA) and visualized under an inverted microscope connected to a confocal scanning unit (Olympus, Tokyo, Japan).

**4.6 ALP activity assay and ALP staining**

The activity of ALP in the supernatant was measured colorimetrically in a reaction mixture (pH 8.8) containing 50 mM Tris-HCl, 10 mM MgCl_2_ and 20 mM p-nitrophenylphosphate with an ALP Activity Detection Kit (Yesen) according to the manufacturer’s protocol (Wang et al., 2013; Hu et al., 2020; Wang et al., 2018). ALP staining was examined using a Violet B Salt Kit (Sigma-Aldrich, Missouri, USA) as reported previously (Wang et al., 2013; Hu et al., 2020; Wang et al., 2018). In brief, osteoblasts were fixed by immersion and rinsed in deionized water. Then, the cells were placed in an ALP stain for 30 min and treated with Mayer’s hematoxylin solution for 10 min. During all the procedures, the samples were protected from light.

**4.7 ELISA**

The BGLAP protein level in the supernatant of osteoblasts was tested using a human or mouse BGLAP ELISA Assay Kit (EIAab, Wuhan, China) with a conventional colorimetric detection method according to the manufacturer’s protocol, as described previously (Wang et al., 2013; Li et al., 2016). Briefly, 100 μl of culture supernatant from osteoblasts was transferred in duplicate into the wells of the BGLAP ELISA plate. Next, the same amount of antibody solution was pipetted into the wells, and the plate was incubated at 37°C for 4 h. Then, each well in the plate was washed three times with PBS.

The collagen I protein content in the supernatant of osteoblasts was examined using a human or mouse Collagen I ELISA Assay Kit (EIAab, Wuhan, China) with a conventional colorimetric detection method (Wang et al., 2013; Li et al., 2016). In brief, 50 μl of culture supernatant from osteoblasts was transferred in duplicate into the wells of the collagen I ELISA plate. Next, the same amount of antibody solution was pipetted into the wells, and the plate was incubated at 37°C for 2 h. Then, each well of the plate was washed three times with PBS.

**4.8 Alizarin red staining**

The osteoblasts in each well were fixed in 70% ice-cold ethanol for 1 h. Then, the plate was gently rinsed with double-distilled H_2_O on a shaking device. The osteoblasts were then stained with 40 mM Alizarin red S (Sigma-Aldrich, Missouri, USA) staining buffer (pH 4.0) for 15 min on a shaking device. Finally, the plate was gently rinsed three times with double-distilled H_2_O for 15 min and then rinsed with PBS for 15 min on a shaking device (Wang et al., 2013; Li et al., 2019).

**4.9 TRAP staining**

TRAP staining was examined with a commercial kit (Sigma-Aldrich, Missouri, USA) according to the manufacturer’s protocol, as described previously (Wang et al., 2013; Li et al., 2016; Li et al., 2019). In brief, the cells or bone slices were stained with working buffer containing naphthol phosphate solution, fast garnet salt, acetate solution and tartrate solution for 1 h in the dark. Then, the samples were fixed in acetone for another 5 min. After fixation, the cells or the bone slices were rinsed three times with double-distilled H_2_O and stained with acid hematoxylin solution. The sample staining was evaluated under a light microscope (Olympus, Tokyo, Japan).

**4.10 Luciferase assay**

Mouse preosteoblasts (MC3T3-E1 cells) were transfected with 50 nM empty vector, WT *Mettl14* 3'UTR, or Mut *Mettl14* 3'UTR for 4 h in Opti-MEM I Reduced-Serum Medium (Gibco, Massachusetts, USA) using Lipofectamine 2000 (Invitrogen, California, USA). Cells were cotransfected with agomir-NC, agomir-103-3p, agomir-103-3p-Mut1, agomir-103-3p-Mut2, antagomir-NC or antagomir-103-3p (RiboBio, Guangzhou, China) at a concentration of 50 nmol/L. The cells were harvested for the luciferase assay 48 h after transfection using a Luciferase Assay Kit (Promega, Wisconsin, USA) according to the manufacturer's instructions (Sun, Cao, Zhang et al., 2015; Sun et al., 2019).

**4.11 RIP assays**

RIP assays were conducted with an RNA-Binding Protein Immunoprecipitation Kit (Millipore, Darmstadt, Germany) as described previously (Ma et al., 2017; Zhang et al., 2019; Han et al., 2019). The primary antibodies included anti- Ago2 antibody (ab32381, Abcam, San Francisco, USA), anti-DGCR8 antibody (ab90579, Abcam, San Francisco, USA) and anti-m^6^A antibody (ab151230, Abcam, San Francisco, USA). At the same time, the total RNA (input control) and IgG (isotype control) were detected for each antibody. The RIP assays were tested via real-time PCR. Immunoprecipitation of DGCR8 or m^6^A was conducted with primary antibodies, and the samples were incubated overnight on a shaking device. After gentle agitation, the immunoprecipitated protein-RNA complexes were evaluated via western blot assays and incubated with proteinase K (Invitrogen, California, USA). The levels of mRNA, miRNA or pri-miRNA were tested via real-time PCR and normalized to input.

**4.12 Western blot assays**

The cells, immunoprecipitation samples or bone specimens were lysed in radioimmunoprecipitation assay buffer (Thermo, Massachusetts, USA) containing a protease inhibitor cocktail (Roche). Protein extracts were boiled with loading buffer (Invitrogen, California, USA). Then, the samples were transferred to polyvinylidene difluoride membranes after separation via SDS-PAGE (Invitrogen, California, USA). The separated protein fractions on the membranes were incubated overnight at 4°C with primary antibodies on a shaking device. The primary antibodies included anti-METTL14 antibody (ab98166, Abcam, San Francisco, USA), anti-DGCR8 antibody (ab90579, Abcam, San Francisco, USA), anti-RUNX2 antibody (ab76956, Abcam, San Francisco, USA), anti-OSX antibody (ab22552, Abcam, San Francisco, USA) and anti-GAPDH antibody (ab8245, Abcam, San Francisco, USA). The secondary antibody was a horseradish peroxidase-conjugated antibody (Jackson, Pennsylvania, USA). The bands were detected through densitometry using Super Signal West substrate (Thermo, Massachusetts, USA) and imaging software (Tanon, Shanghai, China) (Sun, Cao, Zhang et al., 2015; Sun et al., 2019).

**4.13 RNA m^6^A content assays**

The m^6^A content in total RNA was determined with an m^6^A RNA methylation quantification colorimetric kit (Abcam, San Francisco, USA) as reported previously (Ma et al., 2017; Su et al., 2018). Total RNA collected from cells or bone specimens was extracted with TRIzol reagent (Invitrogen, California, USA) and bound to strip wells using a high RNA binding solution. The detected signal was enhanced and then quantified colorimetrically by reading the absorbance in a microplate spectrophotometer (Bio-Rad, California, USA) at a wavelength of 450 nm. The amount of m^6^A reported was proportional to the OD intensity measured.

**4.14 *In vivo* delivery system production and evaluation**

The miR-103-3p *in vivo* delivery system was generated with a miR-103-3p sponge in 293 cells infected with adeno-associated virus and transfected with GFP (RiboBio, Guangzhou, China). The right femurs of C57BL/6J mice were examined with an *in vivo* imaging system (Kodak, Shanghai, China) to track the expression of GFP at 1 to 3 weeks after injection (Xu et al., 2018; Wang, Sun et al., 2019).

**4.15 MicroCT analysis**

The distal femur of each mouse was isolated, fixed in 4% paraformaldehyde and scanned *in vitro* with a microCT system (Siemens, Bavaria, Germany). In brief, the scanning parameters were set as 80 kV and 500 mA. The bone specimens were tested at a resolution of 10 μm with an exposure time of 800 ms/frame. A 3×3×3 mm^3^ cube, which was 2 mm away from the proximal epiphyseal growth plate, was set as the region of interest (ROI) to represent the microstructure of the femur. Three-dimensional reconstruction in the defined ROI was selected to calculate the following parameters: BMD, BV/TV, Tb.N, Tb.Th, Conn.D, Tb.Sp and SMI. For cortical bone, the ROI selected for analysis was of 10% of femoral length in mid-diaphysis of the femur to determine Ct. Th (Wang et al., 2013; Li et al., 2016; Li et al., 2019).

**4.16 Calcein double labeling**

Mice were intraperitoneally injected with 5 mg/kg calcein (Invitrogen, California, USA) at 10 and 3 days before euthanasia. After fixation, dehydration and embedment, the femurs of mice were sliced into 50 μm sections. During the above procedure, the samples were protected from light. Then, bone static parameters (Ob.S/BS, Oc.S/BS, Ob.N/B.Pm and Oc.N/B.Pm) and bone dynamic parameters (MAR and BFR/BS) were calculated with image analysis software under a fluorescence microscope (Olympus, Tokyo, Japan) (Hu et al., 2020; Wang et al., 2018).

**4.17 Masson’s trichrome staining**

Histological sections of femurs were decalcified with EDTA, fixed with paraformaldehyde and embedded in paraffin. Then, the mouse femur sections were cut into 4 μm slices. The bone sections were subjected to modified Masson’s trichrome staining (Sigma-Aldrich, Missouri, USA) following the manufacturer’s instructions as described previously (Wang et al., 2013; Wang et al., 2018).

**4.18 Biomechanical property analysis**

A three-point bending test was conducted to examine the biomechanical properties of the femur with an electromechanical material testing machine (Bose, Massachusetts, USA). The bone specimens were fixed on a bracket at a loading speed of 0.02 mm/s and a span length of 8 mm. The load was exerted on the anterior surface of the diaphyseal midpart, the load was tested, and the deformation data were recorded at 50 Hz. Load deflection curves were used to calculate the maximum load at failure (N), stiffness (N/mm) and elasticity modulus (GPa) (Hu et al., 2020; Wang et al., 2018).

**4.19 Statistical analysis**

Data are expressed as the mean±s.d. Statistical differences among three or more groups were analyzed via one-way analysis of variance (ANOVA) with a Bonferroni *post hoc* test to determine group differences in all numerical data. The significance of differences between two groups was determined with an unpaired, two-tailed Student’s *t* test. The chi-square test was used to examine the comparisons of the categorical data. For statistical correlation, Pearson’s correlation coefficient was used according to requirements. All statistical analyses were performed with SPSS software version 19.0. *P*<0.05 was considered statistically significant.

**References**

Han, J., Wang, J., Yang, X., Yu, H., Zhou, R., Lu, H., … Yang, H. (2019). METTL3 promote tumor proliferation of bladder cancer by accelerating pri-miR221/222 maturation in m^6^A-dependent manner. *Molecular Cancer*, *18*, 110. https://doi: 10.1186/s12943-019-1036-9

Hu, Z., Zhang, L., Wang, H., Wang, Y., Tan, Y., Dang, L., … Zhang, G. (2020). Targeted silencing of miRNA-132-3p expression rescues disuse osteopenia by promoting mesenchymal stem cell osteogenic differentiation and osteogenesis in mice. *Stem Cell Research & Therapy*, *11*, 58. https://doi: 10.1186/s13287-020-1581-6

Li, D., Liu, J., Guo, B., Liang, C., Dang, L., Lu, C., … Zhang, G. (2016). Osteoclast-derived exosomal miR-214-3p inhibits osteoblastic bone formation. *Nature Communications*, *7*, 10872. https://doi: 10.1038/ncomms10872

Li, J., Liu, C., Li, Y., Zheng, Q., Xu, Y., Liu, B., … Li Y. (2019). TMCO1-mediated Ca^2+^ leak underlies osteoblast functions via CaMKII signaling. *Nature Communications*, *10*, 1589. https://doi: 10.1038/s41467-019-09653-5

Ma, J., Yang, F., Zhou, C., Liu, F., Yuan, J., Wang, F., … Sun, S. H. (2017). METTL14 suppresses the metastatic potential of hepatocellular carcinoma by modulating N^6^-methyladenosine-dependent primary microRNA processing. *Hepatology*, *65*, 529-543. https://doi: 10.1002/hep.28885

Su, R., Dong, L., Li, C., Nachtergaele, S., Wunderlich, M., Qing, Y., … Chen, J. (2018). R-2HG exhibits anti-tumor activity by targeting FTO/m^6^A/MYC/CEBPA signaling. *Cell*, *172*, 90-105. https://doi: 10.1016/j.cell.2017.11.031

Sun, Z., Cao, X., Hu, Z., Zhang, L., Wang, H., Zhou, H., … Xie, M. (2015). MiR-103 inhibits osteoblast proliferation mainly through suppressing Cav1.2 expression in simulated microgravity. *Bone*, *76*, 121-128. https://doi: 10.1016/j.bone.2015.04.006

Sun, Z., Cao, X., Zhang, Z., Hu, Z., Zhang, L., Wang, H., … Xie, M. (2015). Simulated microgravity inhibits L-type calcium channel currents partially by up-regulation of miR-103 in MC3T3-E1 osteoblasts. *Scientific Reports*, *5*, 8077. https://doi: 10.1038/srep08077

Sun, Z., Li, Y., Wang, H., Cai, M., Gao, S., Liu, J., … Zhao, J. (2019). MiR-181c-5p mediates simulated microgravity-induced impaired osteoblast proliferation by promoting cell cycle arrested in the G_2_ phase. *Journal of Cellular and Molecular Medicine*, *23*, 3302-3316. https://doi: 10.1111/jcmm.14220

Wang, H., Deng, Q., Lv, Z., Ling, Y., Hou, X., Chen, Z., … Chen, L. (2019). N^6^-methyladenosine induced miR-143-3p promotes the brain metastasis of lung cancer via regulation of VASH1. *Molecular Cancer*, *18*, 181. https://doi: 10.1186/s12943-019-1108-x

Wang, H., Hu, Z., Shi, F., Dong, J., Dang, L., Wang, Y., … Zhang, G. (2018). Osteoblast-targeted delivery of miR-33-5p attenuates osteopenia development induced by mechanical unloading in mice. *Cell Death & Disease*, *9*, 170. https://doi: 10.1038/s41419-017-0210-5

Wang, X., Guo, B., Li, Q., Peng, J., Yang, Z., Wang, A., … Li, Y. (2013). MiR-214 targets ATF4 to inhibit bone formation. *Nature Medicine*, *19*, 93-100. https://doi: 10.1038/nm.3026

Wang, C., Sun, W., Ling, S., Wang, Y., Wang, X., Meng, H., … Peng, J. (2019). AAV-anti-miR-214 prevents collapse of the femoral head in osteonecrosis by regulating osteoblast and osteoclast activities. *Molecular Therapy-Nucleic Acids*, *18*, 841-850. https://doi: 10.1016/j.omtn.2019.09.030

Xu, R., Shen, X., Si, Y., Fu, Y., Zhu, W., Xiao, T., … Jiang, H. (2018). MicroRNA-31a-5p from aging BMSCs links bone formation and resorption in the aged bone marrow microenvironment. *Aging Cell*, *17*, e12794. https://doi: 10.1111/acel.12794

Zhang, J., Bai, R., Li, M., Ye, H., Wu, C., Wang, C., … Lin, D. (2019). Excessive miR-25-3p maturation via N^6^-methyladenosine stimulated by cigarette smoke promotes pancreatic cancer progression. *Nature Communications*, *10*, 1858. https://doi: 10.1038/s41467-019-09712-x


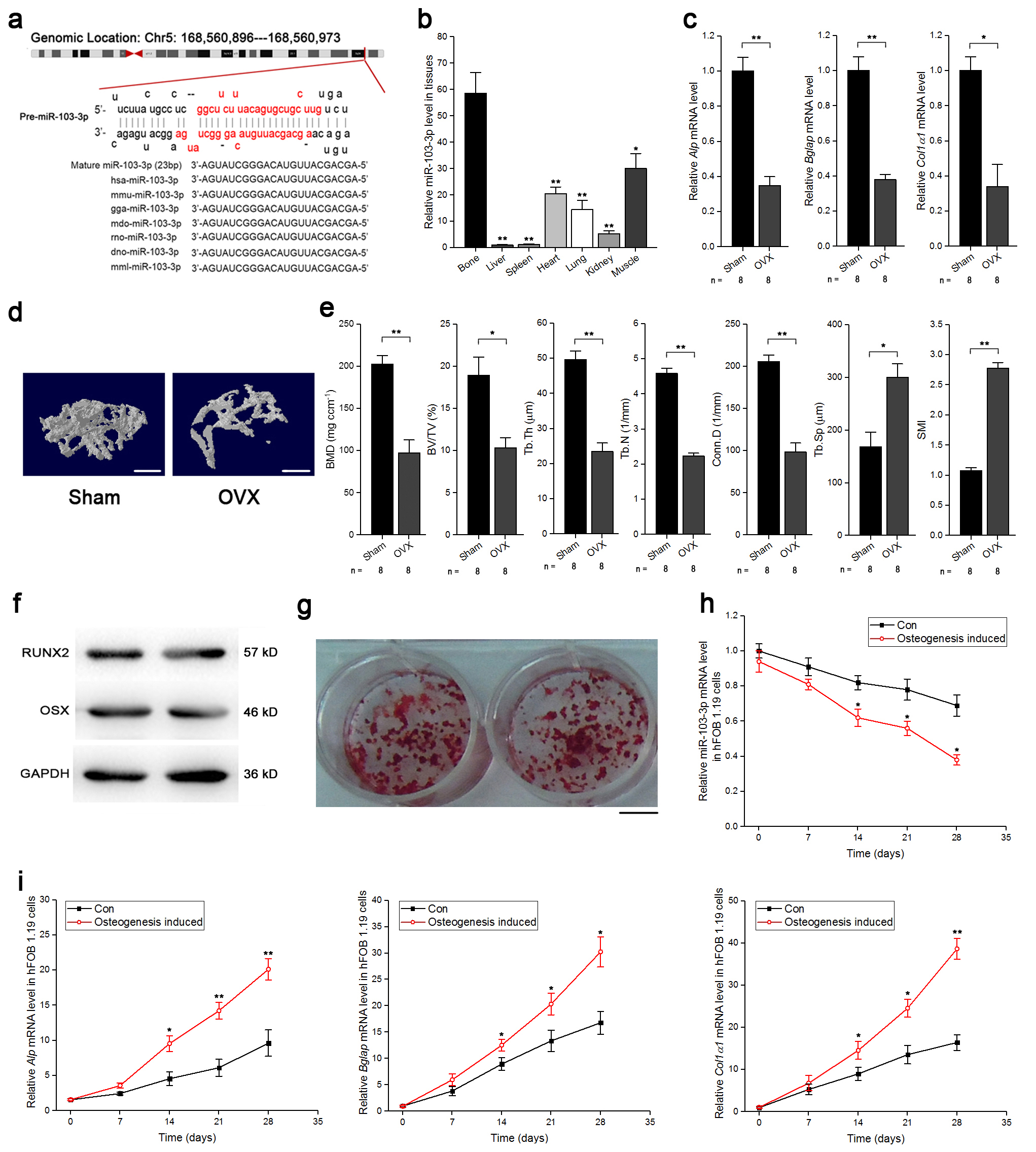


**Figure S1 miR-103-3p level, which is evolutionarily conserved among species, is much higher in bone than in other tissues and negatively correlates with osteoblast differentiation marker genes in hFOB 1.19 cells.** (**a**) Schematic diagram illustrating the genomic localization of the precursor of hsa-miR-103-3p and sequence comparison of mature miR-103-3p among species. (**b**) Real-time PCR analysis of the changes in miR-103-3p level in bone and other tissues from C57BL/6J mice (*n*=4). (**c**) Real-time PCR analysis of the changes in the mRNA levels of the osteoblast differentiation marker genes *Alp* (left), *Bglap* (middle) and *Col1α1* (right) in bone specimens from Sham or OVX mice. The relative mRNA levels were normalized to the mean value of the Sham group. Mouse *Gapdh* mRNA was used as the internal control (*n*=3). (**d**) Representative images showing the three-dimensional trabecular architecture by microCT reconstruction in the distal femurs. Scale bars, 1 mm. (**e**) MicroCT measurement of BMD, BV/TV, Tb.Th, Tb.N, Conn.D Tb.Sp and SMI in the distal femurs of Sham or OVX mice. (**f**) Western blot analysis of the amount of Runt-related transcription factor 2 (RUNX2) and Osterix (OSX) protein in primary mouse osteoblasts (n=3). (**g**) Alizarin red staining of calcium deposition in primary mouse in osteogenic medium for 21 days. Scale bar, 10 mm. (**h**) Real-time PCR analysis of miR-103-3p level during osteoblast maturation in hFOB 1.19 cells (*n*=3). (**i**) Real-time PCR analysis of the changes in the mRNA levels of the osteoblast differentiation marker genes *Alp* (left), *Bglap* (middle) and *Col1α1* (right) during osteoblast maturation in hFOB 1.19 cells (*n*=3). The *n* value for each group is indicated at the bottom of each bar in the graphs. All data are the mean$\pm$s.d. ^*^*P* < 0.05, ^**^*P* < 0.01. One-way ANOVA with a *post hoc* test was performed, and the significance of differences between two groups was determined with Student’s *t* test.


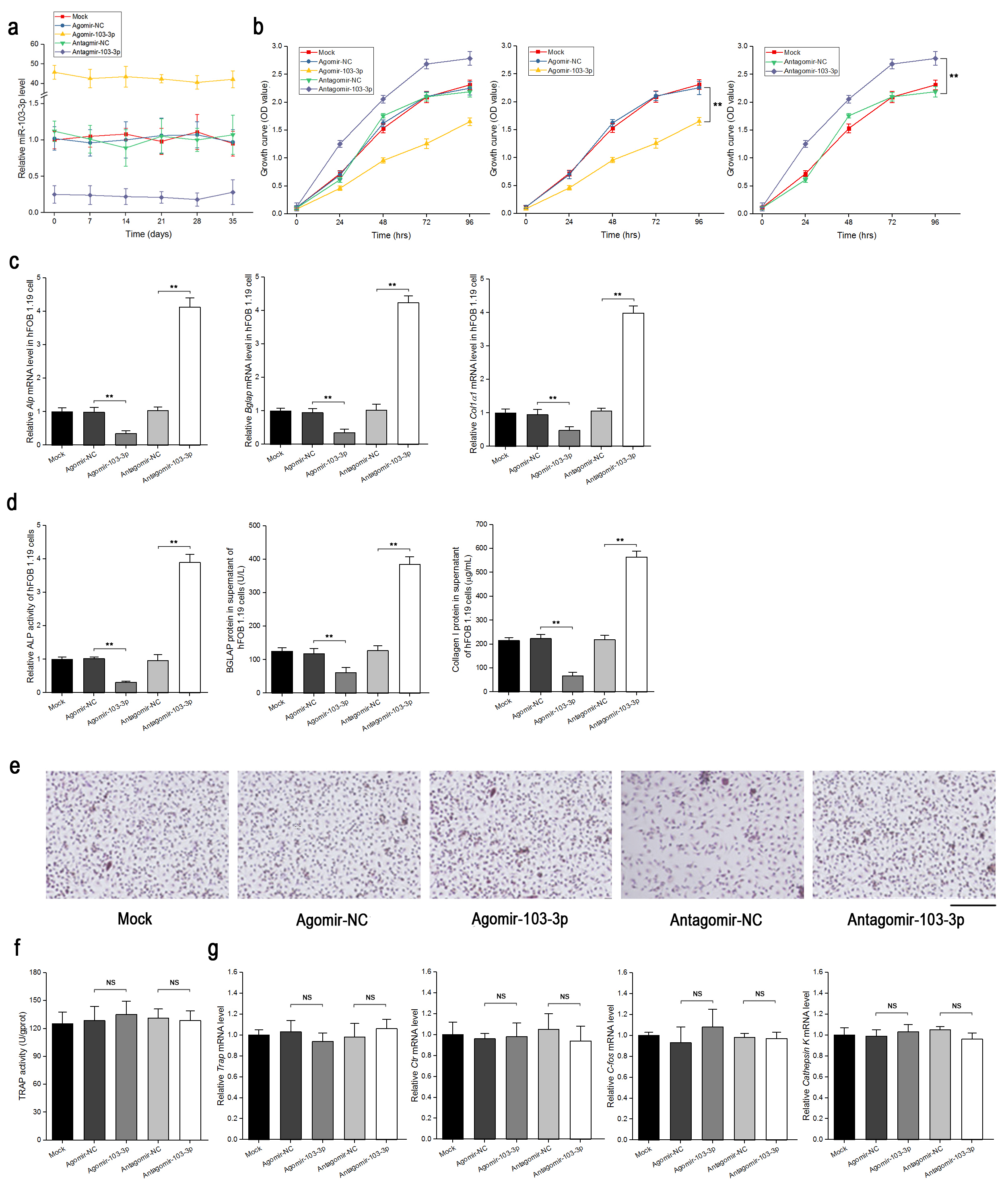


**Figure S2 The effects of miR-103-3p on the activity of hFOB 1.19 cells and osteoclasts.** (**a**) Real-time PCR analysis of the time course of changes in miR-103-3p level in primary mouse osteoblasts after treatment with agomir-103-3p, antagomir-103-3p or the corresponding negative controls in osteogenic medium (ascorbic acid, dexamethasone and β-sodium glycerophosphate) (n=3). (**b**) WST-8 assay of changes in cell growth in hFOB 1.19 cells at 24-96 h after treatment with 300 μM agomir-103-3p, antagomir-103-3p or the corresponding negative controls (n=3). (**c**) Real-time PCR analysis of the changes in the mRNA levels of the osteoblast differentiation marker genes *Alp* (left), *Bglap* (middle) and *Col1α1* (right) in hFOB 1.19 cells after treatment with 300 μM agomir-103-3p, antagomir-103-3p or the corresponding negative controls for 48 h (n=3). (**d**) ALP activity (left) and the amount of BGLAP protein (middle) and collagen I (right) in the supernatant of hFOB 1.19 cells after treatment with 300 μM agomir-103-3p, antagomir-103-3p or the corresponding negative controls for 48 h (n=3). (**e**) Representative images of osteoclasts subjected to tartrate-resistant acid phosphatase (TRAP) staining after treatment with 300 μM agomir-103-3p, antagomir-103-3p or the corresponding negative controls for 48 h (n=3). Scale bar, 500 μm. (**f**) The activity of TRAP from each group was quantified using test kits (n=3). (**g**) Real-time PCR analysis of the mRNA levels of the osteoclast activity marker genes *Trap*, *Ctr*, *C-fos* and *Cathepsin K* in osteoclasts after treatment with 300 μM agomir-103-3p, Antagomir-103-3p or the corresponding negative controls for 48 h (n=3). All data are presented as the mean$\pm$s.d. ^**^*P* < 0.01. One-way ANOVA with a *post hoc* test was performed, and the significance of differences between two groups was determined with Student’s *t* test.


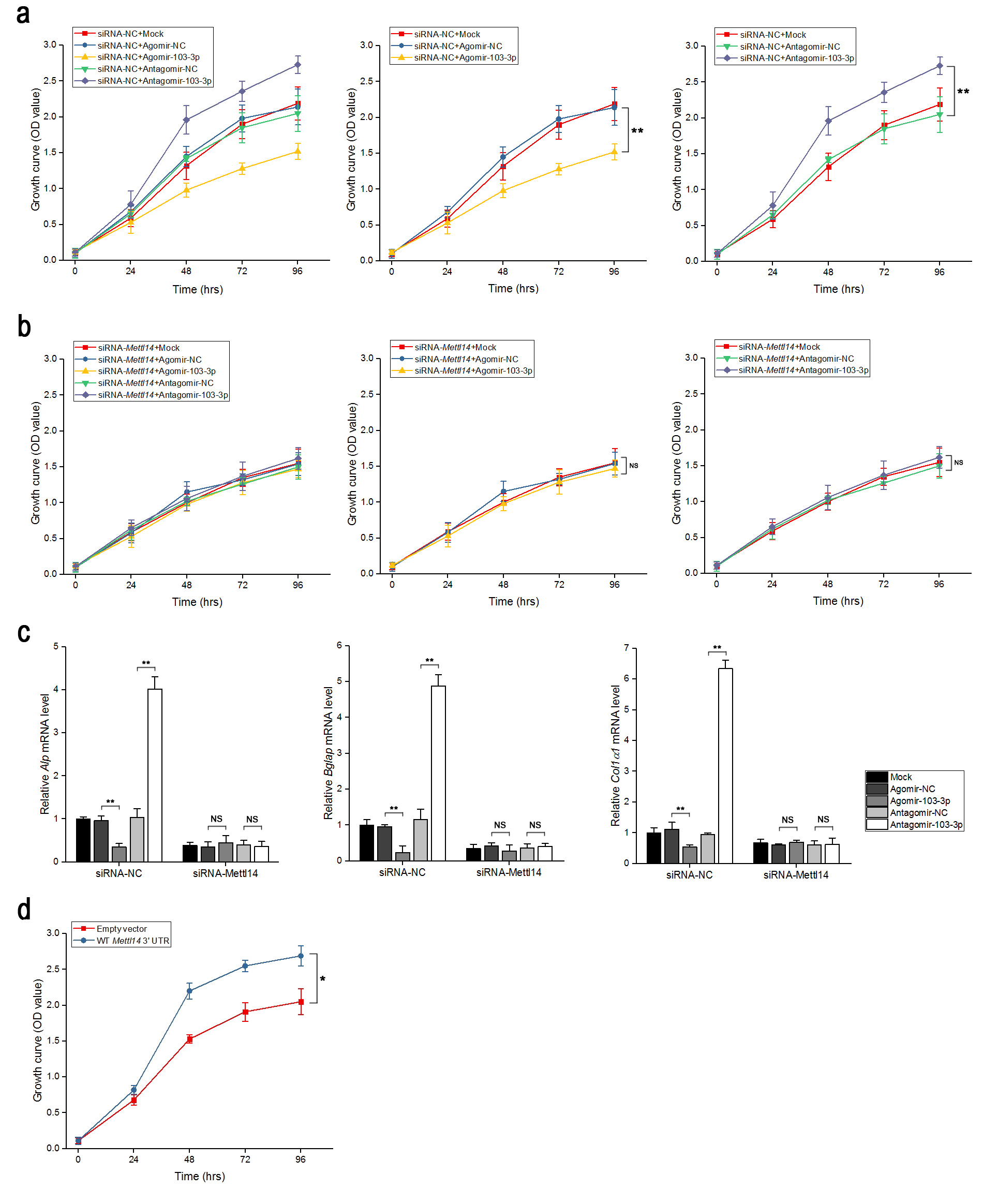


**Figure S3 The effect of miR-103-3p on the function of osteoblasts is *Mettl14* dependent.** (**a**) and (**b**) WST-8 assay analysis of changes in cell growth in primary mouse osteoblasts at 24-96 h after silencing *Mettl14* with siRNA-*Mettl14* under treatment with agomir-103-3p, antagomir-103-3p or the corresponding negative controls (n=3). The results from the siRNA-NC and a mock transfection are also shown. (**c**) Real-time PCR analysis of the changes in the mRNA levels of the osteoblast differentiation marker genes *Alp* (left), *Bglap* (middle) and *Col1α1* (right) in primary mouse osteoblasts after silencing *Mettl14* with siRNA-*Mettl14* under treatment with agomir-103-3p, antagomir-103-3p or the corresponding negative controls (n=3). The results from siRNA-NC and mock transfection are also shown. (**d**) WST-8 assay of changes in cell growth in primary mouse osteoblasts at 24-96 h after blockade of miR-103-3p binding to *Mettl14* by overexpression of WT *Mettl14* 3’UTR (n=3). All data are presented as the mean$\pm$s.d. ^*^*P* < 0.05, ^**^*P* < 0.01. One-way ANOVA with a *post hoc* test was performed, and the significance of differences between two groups was determined with Student’s *t* test.


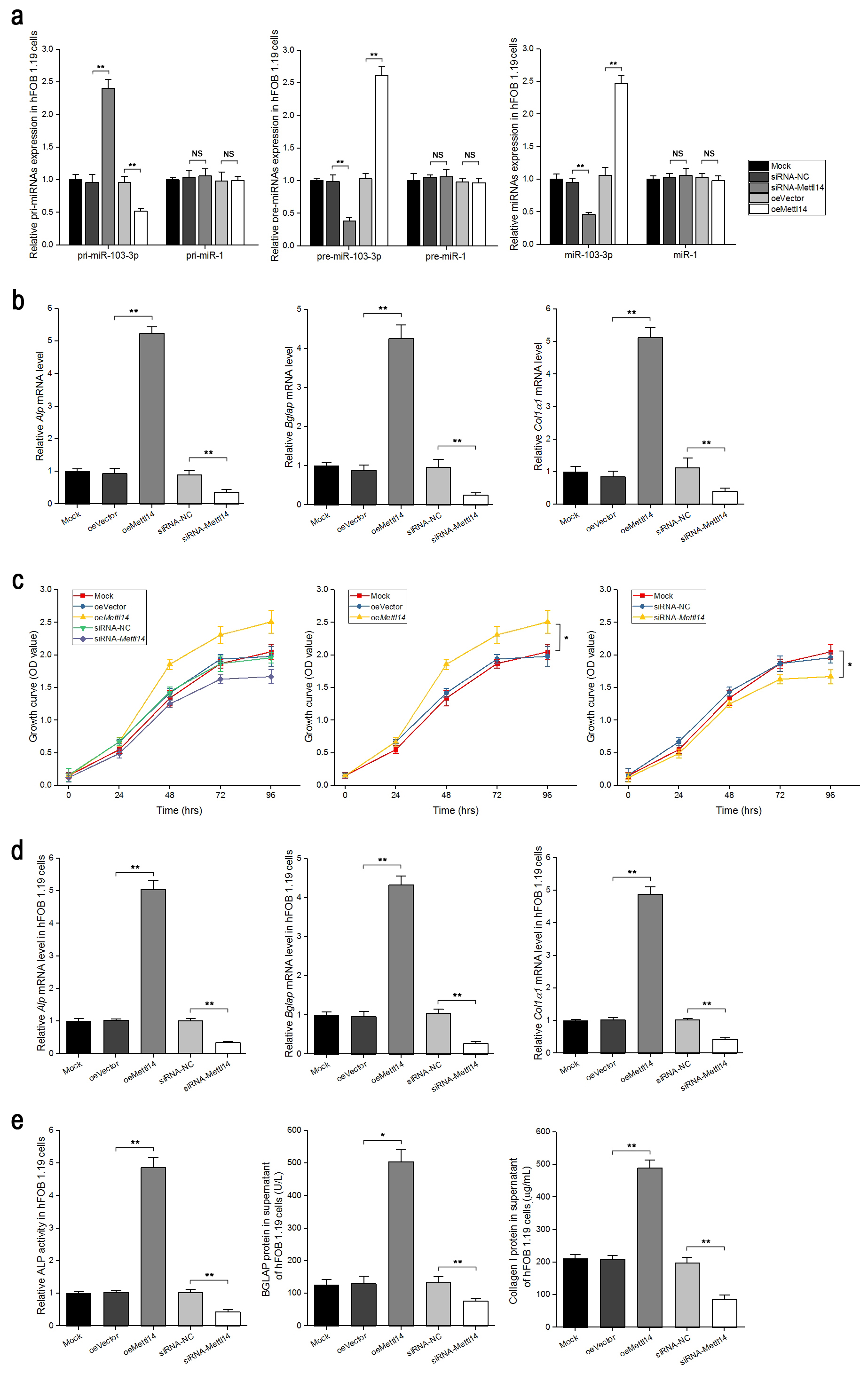


**Figure S4 METTL14-dependent m^6^A methylation regulates the processing of miR-103-3p and osteoblast activity *in vitro*.** (**a**) Real-time PCR analysis of pri-miR-103-3p (left), pre-miR-103-3p (middle) and miR-103-3p (right) in METTL14 knockdown and overexpression hFOB 1.19 cells (n=3). (**b**) Real-time PCR analysis of the changes in the mRNA levels of the osteoblast differentiation marker genes *Alp* (left), *Bglap* (middle) and *Col1α1* (right) in primary mouse osteoblasts after treatment with oe*Mettl14*, siRNA-*Mettl14* or the corresponding negative controls (n=3). (**c**) WST-8 assay of changes in cell growth at 24-96 h after treatment with oe*Mettl14*, siRNA-*Mettl14* or the corresponding negative controls in hFOB 1.19 cells (n=3). (**d**) Real-time PCR analysis of the changes in the mRNA levels of the osteoblast differentiation marker genes *Alp* (left), *Bglap* (middle) and *Col1α1* (right) in hFOB 1.19 cells after treatment with oe*Mettl14*, siRNA-*Mettl14* or the corresponding negative controls (n=3). (**e**) ALP activity (left) and the amount of BALP protein (middle) and collagen I (right) in the supernatant of hFOB 1.19 cells after treatment with oe*Mettl14*, siRNA-*Mettl14* or the corresponding negative controls (n=3). All data are presented as the mean$\pm$s.d. ^*^*P* < 0.05, ^**^*P* < 0.01. One-way ANOVA with a *post hoc* test was performed, and the significance of differences between two groups was determined with Student’s *t* test.


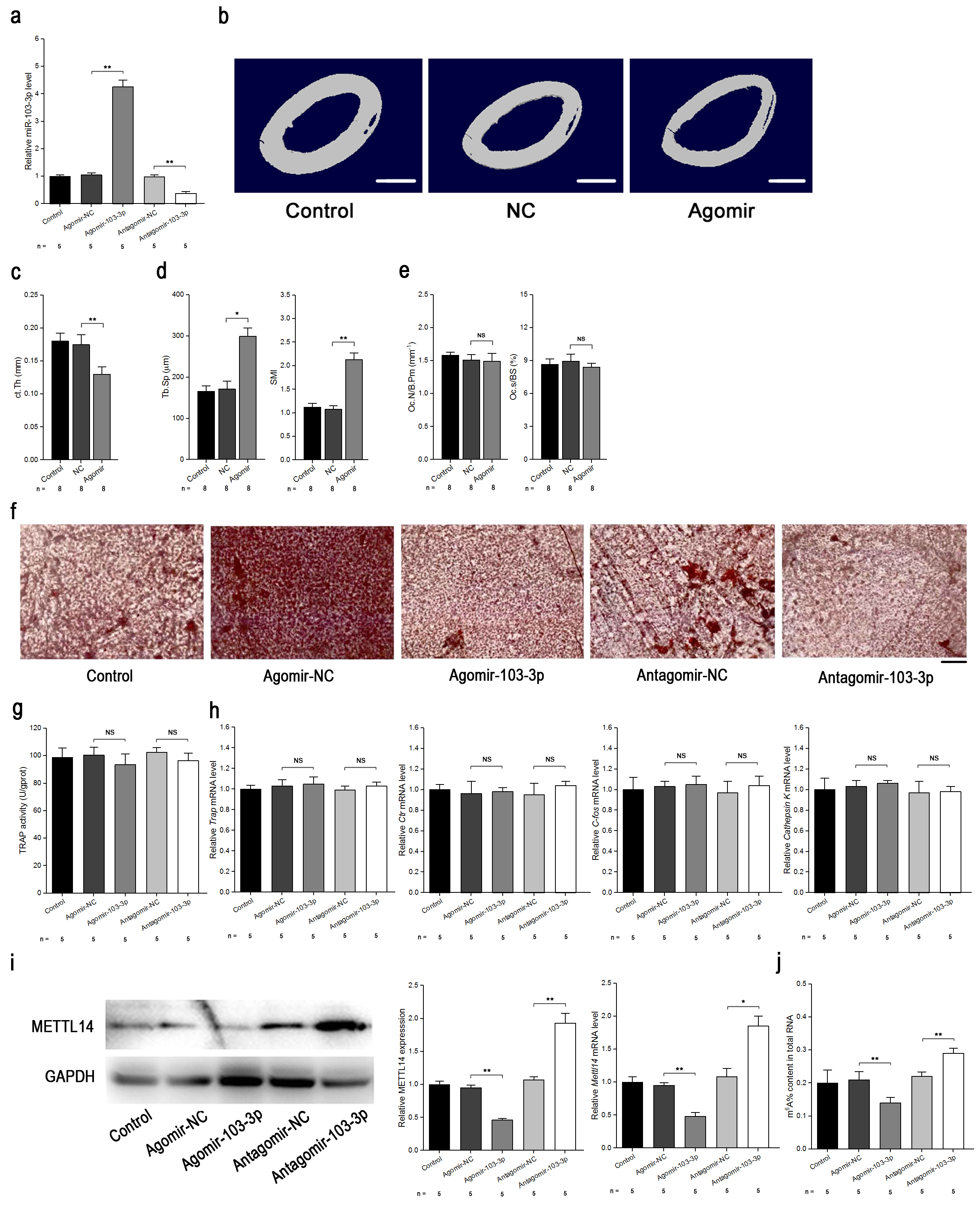


**Figure S5 miR-103-3p targets *Mettl14* to inhibit bone formation *in vivo*.** (**a**) Real-time PCR analysis of miR-103-3p levels in bone specimens from mice after treatment with agomir-103-3p, antagomir-103-3p or the corresponding negative controls. (**b**) Representative images showing the three-dimensional cortical bone in the femurs by microCT reconstruction. Scale bars, 1 mm. (**c**) MicroCT measurement of cortical thickness (Ct.Th) in the femurs of mice after treatment with agomir-103-3p or its negative control. (**d**) MicroCT measurement of Tb.Sp and SMI in the distal femurs of mice after treatment with agomir-103-3p or its negative control. (**e**) Double calcein labeling analysis of dynamic bone histomorphometric parameters (Oc.N/B.Pm and Oc.s/BS) in the distal femurs of mice after treatment with agomir-103-3p or its negative control. (**f**) Representative images of osteoclasts subjected to TRAP staining after treatment with agomir-103-3p, antagomir-103-3p or the corresponding negative controls (n=3). Scale bar, 10 μm. (**g**) The activity of TRAP in each group was quantified using test kits. (**h**) Real-time PCR analysis of the mRNA levels of the osteoclast activity marker genes *Trap*, *Ctr*, *C-fos* and *Cathepsin K* in mouse bone specimens after treatment with agomir-103-3p, Antagomir-103-3p or the corresponding negative controls. (**i**) The effect of agomir-103-3p, antagomir-103-3p or the corresponding negative controls on the amount of METTL14 protein (left and middle) and *Mettl14* mRNA levels (right) in mouse bone specimens. (**j**) The effects of agomir-103-3p, antagomir-103-3p or the corresponding negative controls on the m^6^A content of total RNA in mouse bone specimens. The *n* value for each group is indicated at the bottom of each bar in the graphs. All data are presented as the mean$\pm$s.d. ^*^*P* < 0.05, ^**^*P* < 0.01. One-way ANOVA with a *post hoc* test was performed, and the significance of differences between two groups was determined with Student’s *t* test.

**
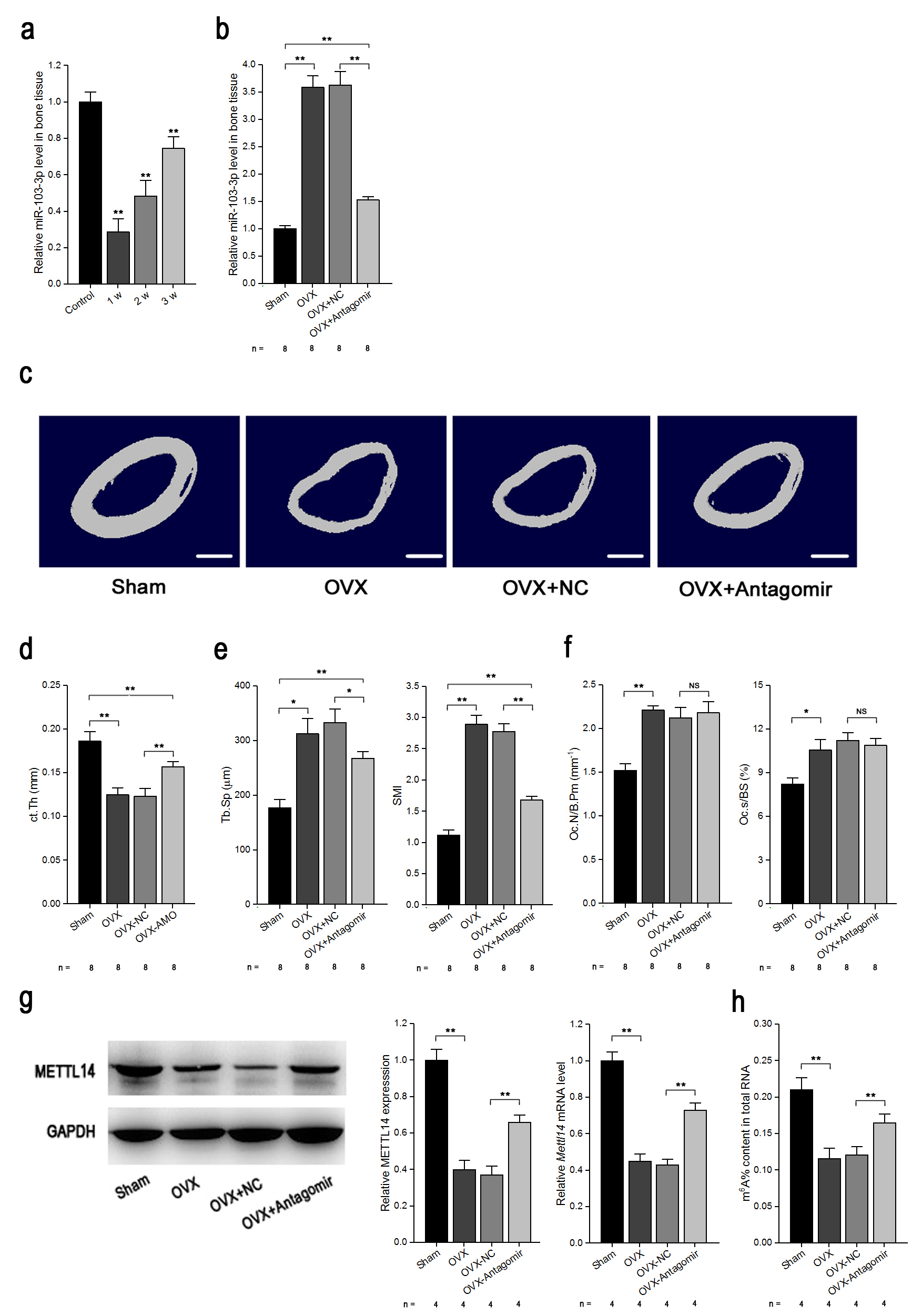
**

**Figure S6 The effect of therapeutic inhibition of miR-103-3p on mice with OVX-induced osteoporosis.** (**a**) Real-time PCR analysis of miR-103-3p levels in bone specimens from mice at 1 to 3 weeks after treatment with antagomiR-103-3p (n=3). (**b**) Real-time PCR analysis of miR-103-3p levels in whole bone tissue collected from OVX mice before treatment (Sham), OVX mice without treatment (OVX), and OVX mice treated with antagomir-NC (OVX+NC) and antagomiR-103-3p (OVX+antagomir). (**c**) Representative images showing the three-dimensional cortical bone in the femurs by microCT reconstruction. Scale bars, 1 mm. (**d**) MicroCT measurement of cortical thickness (Ct.Th) in the femurs collected from the Sham, OVX, OVX+NC and OVX+Antagomir groups. (**e**) MicroCT measurement of Tb.Sp and SMI in whole bone tissue collected from the Sham, OVX, OVX+NC and OVX+Antagomir groups. (**f**) Double calcein labeling analysis of dynamic bone histomorphometric parameters (Oc.N/B.Pm and Oc.s/BS) in whole bone tissue collected from the Sham, OVX, OVX+NC and OVX+Antagomir groups. (**g**) The effect of antagomir-103-3p or its negative control on the amount of METTL14 protein (left and middle) and *Mettl14* mRNA levels (right) in whole bone tissue collected from the Sham, OVX, OVX+NC and OVX+antagomir groups. (**h**) The effect of antagomir-103-3p or its negative control on the m^6^A content of total RNA in whole bone tissue collected from the Sham, OVX, OVX+NC and OVX+antagomir groups. The *n* value for each group is indicated at the bottom of each bar in the graphs. All data are presented as the mean$\pm$s.d. ^*^*P* < 0.05, ^**^*P* < 0.01. One-way ANOVA with a *post hoc* test was performed, and the significance of differences between two groups was determined with Student’s *t* test.

**Figure S7 Correlations between the miR-103-3p/METTL14/m^6^a axis and bone formation capacity in human bone specimens, mouse bone specimens and primary mouse osteoblasts.** (**a**) METTL14 protein and *METTL14* mRNA (first three) levels and the m^6^A content in total RNA (last) in bone specimens from patients. (**b**) Correlation analysis between the *METTL14* mRNA level and the m^6^A content in total RNA in bone specimens from patients. (**c**) Correlation analysis between the miR-103-3p level and *METTL14* mRNA level (left) and the m^6^A content in total RNA (right) in bone specimens from patients. (**d**) Correlation analysis between the *METTL14* mRNA level and *ALP* (left), *BGLAP* (middle) and *COL1α1* (right) mRNA levels in bone specimens from patients. (**e**) Correlation analysis between the m^6^A content in total RNA and *ALP* (left), *BGLAP* (middle) and *COL1α1* (right) mRNA levels in bone specimens from patients. (**f**) METTL14 protein and *Mettl14* mRNA (first three) levels and the m^6^A content in total RNA (last) in bone specimens from Sham or OVX mice. (**g**) Correlation analysis between the *Mettl14* mRNA level and the m^6^A content in total RNA in bone specimens from Sham or OVX mice. (**h**) Correlation analysis between the miR-103-3p level and *Mettl14* mRNA level (left) and the m^6^A content in total RNA (right) in bone specimens from Sham or OVX mice. (**i**) Correlation analysis between the *Mettl14* mRNA level and *Alp* (left), *Bglap* (middle) and *Col1α1* (right) mRNA levels in bone specimens from Sham or OVX mice. (**j**) Correlation analysis between the m^6^A content in total RNA and *Alp* (left), *Bglap* (middle) and *Col1α1* (right) mRNA levels in bone specimens from Sham or OVX mice. (**k**) Real-time PCR analysis of *Alp*, *Bglap* and *Col1α1* mRNA levels (first three) and the miR-103-3p level (last) in primary mouse osteoblasts during osteoblast maturation (*n*=3). (**l**) The METTL14 protein and *Mettl14* mRNA (first three) levels and the m^6^A content in total RNA (last) in primary mouse osteoblasts during osteoblast maturation (*n*=3). (**m**) Schematic model of the role and underlying mechanism of the miR-103-3p/METTL14/m^6^a axis in suppressing osteoblast activity and aggravating postmenopausal osteoporosis. The *n* value for each group is indicated at the bottom of each bar in the graphs. All data are the mean$\pm$s.d. ^*^*P* < 0.05, ^**^*P* < 0.01. One-way ANOVA with a *post hoc* test was performed, and the significance of differences between two groups was determined with Student’s *t* test. For statistical correlation, Pearson’s correlation coefficient was used.

**
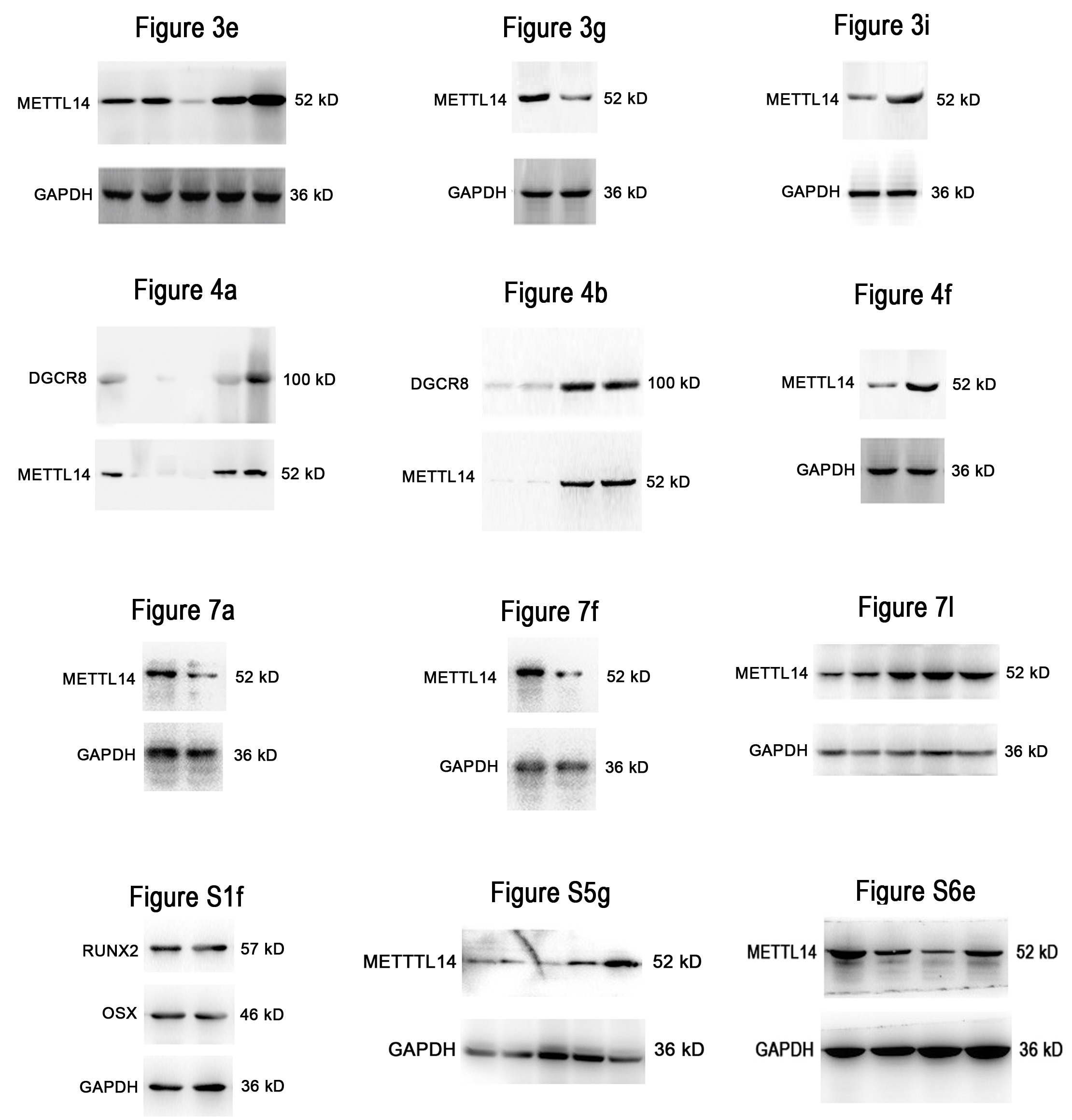
**

**Figure S8 The uncropped Western blot scans.**

| **Table S1. Clinical features of fracture patients involved in bone specimens analysis** | | | | | | | |
| --- | --- | --- | --- | --- | --- | --- | --- |
| **Patient** | **Age** | **Gender** | **Menopause age** | **T score for BMD at Lumbar Spine** | **Serum BALP (U/L)** | **Diagnosis** | **Surgical operation** |
| 1 | 75 | Female | 50 | -1.25 | 13.5 | Femoral Neck Fracture (Left) | Total Hip Replacement |
| 2 | 73 | Female | 53 | -1.53 | 12.7 | Proximal Femur Fracture (Right) | Femoral Head Replacement |
| 3 | 72 | Female | 52 | -2.87 | 9.1 | Femoral Neck Fracture (Right) | Total Hip Replacement |
| 4 | 68 | Female | 50 | -3.02 | 8.6 | Femoral Neck Fracture (Right) | Total Hip Replacement |
| 5 | 67 | Female | 49 | -0.85 | 25.3 | Femoral Neck Fracture (Left) | Total Hip Replacement |
| 6 | 85 | Female | 53 | -2.12 | 11.2 | Proximal Femur Fracture (Right) | Femoral Head Replacement |
| 7 | 86 | Female | 48 | -2.71 | 13.5 | Proximal Femur Fracture (Right) | Femoral Head Replacement |
| 8 | 89 | Female | 55 | -2.34 | 10.3 | Proximal Femur Fracture (Right) | Total Hip Replacement |
| 9 | 73 | Female | 49 | -1.06 | 17.3 | Femoral Neck Fracture (Left) | Femoral Head Replacement |
| 10 | 67 | Female | 48 | -0.56 | 24.6 | Proximal Femur Fracture (Right) | Total Hip Replacement |
| 11 | 66 | Female | 52 | -2.68 | 14.1 | Femoral Neck Fracture (Left) | Total Hip Replacement |
| 12 | 81 | Female | 47 | -1.36 | 12.3 | Proximal Femur Fracture (Left) | Total Hip Replacement |
| 13 | 80 | Female | 50 | -2.77 | 15.3 | Proximal Femur Fracture (Right) | Total Hip Replacement |
| 14 | 83 | Female | 52 | -2.67 | 14.7 | Femoral Neck Fracture (Left) | Total Hip Replacement |
| 15 | 69 | Female | 51 | -1.02 | 19.8 | Femoral Neck Fracture (Right) | Femoral Head Replacement |
| 16 | 73 | Female | 49 | -1.08 | 11.8 | Femoral Neck Fracture (Right) | Femoral Head Replacement |
| 17 | 71 | Female | 48 | -2.84 | 13.9 | Femoral Neck Fracture (Right) | Femoral Head Replacement |
| 18 | 80 | Female | 50 | -1.66 | 12.0 | Femoral Neck Fracture (Left) | Total Hip Replacement |
| 19 | 76 | Female | 52 | -3.04 | 8.1 | Proximal Femur Fracture (Left) | Total Hip Replacement |
| 20 | 77 | Female | 46 | -1.56 | 16.6 | Femoral Neck Fracture (Left) | Total Hip Replacement |
| 21 | 82 | Female | 53 | -2.78 | 14.1 | Femoral Neck Fracture (Left) | Total Hip Replacement |
| 22 | 79 | Female | 51 | -2.66 | 15.3 | Femoral Neck Fracture (Left) | Total Hip Replacement |
| 23 | 67 | Female | 49 | -2.13 | 18.9 | Proximal Femur Fracture (Right) | Total Hip Replacement |
| 24 | 74 | Female | 48 | -1.53 | 13.8 | Proximal Femur Fracture (Left) | Femoral Head Replacement |

| **Table S2. The comparisons of the clinical characteristics between T>−2.5 and T≤−2.5 groups**   \|  \| **T>−2.5 group** \| **T≤−2.5 group** \| ***t* / *χ^2^* value** \| ***P* value** \| \| --- \| --- \| --- \| --- \| --- \| \| **Case number** \| 14 \| 10 \|  \|  \| \| **Mean age (years) ^a^** \| 75.00±6.80 \| 76.30±6.78 \| -0.462 \| 0.649 \| \| **Mean menopause age (years) ^a^** \| 49.79±2.49 \| 50.80±1.75 \| -1.106 \| 0.281 \| \| **Injured limb (right/left)^b^** \| 7/7 \| 7/7 \| 0.000 \| 1.000 \|   ^a^Examined by the two-tailed Student’s *t* test. ^b^Examined by the chi-square test.  **Table S3. The oligonucleotide sequences used in the study** | | |
| --- | --- | --- | --- | --- | --- | --- | --- | --- | --- | --- | --- | --- | --- | --- | --- | --- | --- | --- | --- | --- | --- | --- | --- | --- | --- | --- | --- |
| **The oligonucleotide name** | **Sense sequences (5'-3')** | **Antisense sequences (5'-3')** |
| Agomir-103-3p | AGCCGCCUUGUACAGGGCUAUGA | UCAUAGCCCUGUACAAUGCUGCU |
| Agomir-NC | UUUGUACUACACAAAAGUACUG | CAGUACUUUUGUGUAGUACAAA |
| Agomir-103-3p-Mut1 | CCGUCGUAUGUACAGGGCUAUGA |  |
| Agomir-103-3p-Mut2 | UCGUCGUAACAUGUCCCGAUACU |  |
| Antagomir-103-3p | UCAUAGCCCUGUACAAUGCUGCU |  |
| Antagomir-NC | GUCAUGAAAACACAUCAUGUUU |  |
| WT *Mettl14* 3'UTR | UUGCUUUAGUUUCUCAUGCUGCC |  |
| Mut *Mettl14* 3'UTR | UUGCUUUAGUUUCUCUACGACGC |  |
| siRNA-*Mettl14* | GGCUAAAGGAUGAGUUAAUTT | AUUAACUCAUCCUUUAGCCTT |
| siRNA-NC | UUCUCCGAACGUGUCACGUTT | ACGUGACACGUUCGG AGAATT |

| **Table S4. The primer sequences used for real-time PCR** | | |
| --- | --- | --- |
| **Gene** | **Forward primers (5’-3’)** | **Reverse primers (5’-3’)** |
|  |  |  |
| Human *ALP* | GGACCATTCCCACGTCTTCAC | CCTTGTAGCCAGGCCCATTG |
| Human *BGLAP* | CTCACACTCCTCGCCCTATTGG | GTAGCGCCTGGGTCTCTTCACT |
| Human *COL1*α*1* | CGATGGATTCCAGTTCGAGTATG | TGTTCTTGCAGTGGTAGGTGATG |
| Human *METTL14* | GTCTTAGTCTTCCCAGGATTGTTT | AATTGATGAGATTGCAGCACC |
| Human *GAPDH* | CGGATTTGGTCGTATTGGG | CTGGAAGATGGTGATGGGATT |
| Mouse *Alp* | CGTGGGCATTGTGACTACC | CTGGTGGCATCTCGTTATCC |
| Mouse *Bglap* | GACAAGTCCCACACAGCAACT | GGACATGAAGGCTTTGTCAGA |
| Mouse *Col1α1* | GACATGTTCAGCTTTGTGGACCTC | GGGACCCTTAGGCCATTGTGTA |
| Mouse *Mettl14* | TCTGGAAAACTGCCTTTGGAT | AAATGCTGGACCTGGGATGAT |
| Mouse *Trap* | GTGGAAGCCTCTGGAAAATC | CTCCTCCCTCACACCCGTTA |
| Mouse *Ctr* | TGCAGACAACTCTTGGTTGG | TCGGTTTCTTCTCCTCTGGA |
| Mouse *C-fos* | TCCAGATTGCTGGACAATGA | CCCGTCTTGGCATACATCTT |
| Mouse Ctsk | GAAGAAGACTCACCAGAAGCAG | TCCAGGTTATGGGCAGAGATT |
| Mouse *Gapdh* | CCTCTGACTTCAACAGCGAC | TCCTCTTGTGCTCTTGCTGG |
| hus-miR-103-3p | ACACTCCAGCTGGGAGCAGCATTGTAC | TGGTGTCGTGGAGTCG |
| mmu-miR-103-3p | ACACTCCAGCTGGGAGCAGCATTGTAC | TGGTGTCGTGGAGTCG |
| mmu-pre-miR-103-3p | GTCTCAATGCCTTCATAGCCCTGTAT | AGCCGAGGGCAGTAAGAA |
| mmu-pri-miR-103-3p | GAAGAAGACTCACCACCAGCTGGGT | TGGCATACATCTATGTATG |
| mmu-pre-miR-1 | GAAACATACTTCTTTATATGCCCAT | ATTGCCCTATGTATGAAGAAATGTAAGGTAT |
| mmu-pri-miR-1 | GCGTCCCGGGGTCTTGGAACTG | GCCGCCTGGCTGGCTGTCG |
| mmu-pri-miR-let-7e | TGTGGGTCCGTGTCGGGG | CTGAGTGGGGCTGGGGGTC |
| *U6* | CTCGCTTCGGCAGCACA | AACGCTTCACGAATTTGCGT |
